# Drop Dilution Enables the Use of PEG-Derived Detergents for Membrane Protein Purification

**DOI:** 10.1021/acsomega.5c09173

**Published:** 2025-11-14

**Authors:** Katharina Alker, Shweta Singh, Arun K. Vinodakrishnan, Florian Lindemann, Rasmus Linser, Ram Singh, Abhishek K. Singh, Leonhard H. Urner

**Affiliations:** † 14311 TU Dortmund University, Department of Chemistry and Chemical Biology, Otto-Hahn-Str. 6, 44227 Dortmund, Germany; ‡ Department of Applied Chemistry, 231511 Delhi Technological University, Delhi 110042, India; § 9166 Freie Universität Berlin, Institute of Chemistry and Biochemistry, Takustr. 3, 14195 Berlin, Germany

## Abstract

PEG detergents are important tools in the biophysical characterization of membrane protein whose utility is often limited by their intrinsic denaturing properties. This work addresses the question of whether changing the linker between PEG headgroup and nonpolar tail can modulate the denaturing properties of these detergents. To address this question, herein, we introduce the modular architecture of PEG550 detergents and explore its utility for protein purification from membranes and detergent exchange. Our results indicate that PEG550 detergents cannot efficiently solubilize proteins from lysed bacterial membranes. Varying the linker cannot eliminate the denaturing properties that PEG550 detergents can have on a protein during extraction and affinity purification. Interestingly, we find that PEG550 detergents can preserve the secondary structure and activity of the model membrane protein vitamin B12 transporter as good as the reference detergent n-dodecyl-β-D-maltoside following detergent exchange via drop dilution. Our findings clarify that denaturing properties of PEG550 detergents depend on both their chemical structure and the detergent exchange method with which proteins and detergents are brought together. Our drop dilution conditions are representative of those frequently employed in the biophysical characterization of membrane proteins. We anticipate PEG550 detergents will deliver a starting point for the optimization of sample properties in the biophysical characterization of membrane proteins.

## Graphical abstract

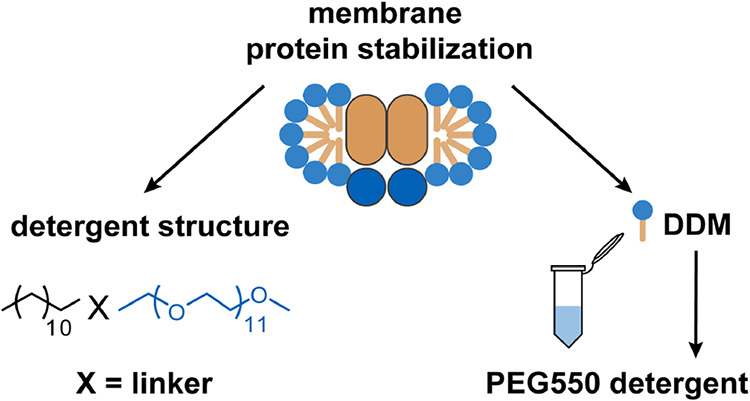

## Introduction

Research into membrane proteins has been at the center of biomedical science for decades because of its profound impact on human health.^1^ Membrane proteins fulfill a variety of important functions, including cell–cell recognition, membrane transport, signal transduction, cell adhesion and mobility.^2^ Approximately 25% of the human genome encodes for membrane proteins, underscoring their biological importance. Dysregulation or dysfunction of membrane proteins is associated with numerous diseases, including various cancers, viral infections and neurodegenerative diseases, such as Alzheimer’s and Parkinson’s disease. As a result, over 50% of all therapeutic drugs target these membrane-associated biomacromolecules, highlighting their critical role in drug discovery.^3–5^

Nonionic detergents are key components in membrane protein research.^6^ Detergents are amphiphilic molecules that have both hydrophilic and hydrophobic domains, which enable them to form micelles in aqueous solution. Detergents can enable the extraction, purification and stabilization of membrane proteins in micellar solutions, making them crucial for structural and biochemical characterization.^7,8^ However, the choice of detergent is a critical factor in this process as it determines the efficiency of solubilization and preservation of structural and functional integrity of the target protein. From a detergent structure point of view, enormous efforts have been made to improve the efficacy of detergents in stabilizing membrane proteins. Chemical design concepts include the fusion of detergent head groups and/or tails,^9^ modular detergents,^10,11^ detergent mixtures,^12^ polymerizable detergents,^13^ detergent fluorination,^14^ detergent asymmetry,^15^ detergent rigidity,^16^ peptide-based detergents,^17^ and living detergents.^18^ Finding new concepts to optimize detergents for protein purification can deliver enabling steps for downstream applications, such as structural studies, functional assays and drug screening.^7,19,20^

Poly­(ethylene glycol) (PEG) is widely regarded as a universal hydrophilic entity in detergent chemistry due to its excellent water solubility, biocompatibility and flexibility in molecular design. In addition, the adjustable length of the PEG chains allows precise control over micelle size and aggregation behavior.^11^ Modulating the size and composition of PEG head groups unlocked possibilities for applications in membrane protein research, such as enhancing protein activity,^9^ charge reduction in native mass spectrometry,^21^ improved membrane protein stabilization,^22^ and cell membrane disruption.^6^ Various PEG detergent architectures have been introduced, including asymmetric hybrid detergents with covalent combination of tetraethylene glycol (E4) with other nonionic head groups,^9^ PEG detergents with variations in the number of ethylene glycol repetition units (C8E4, Brij 98),^21^ PEG-transition linker detergents with the use of oligoethylene glycol fragments as linkers between detergent headgroup and tail (T2-DDM),^22^ variations in the structure of nonpolar tails (Triton X-100 and Brij 98),^6^ and hybrid “dimer” detergents consisting of two heads and two tails (8–12M-E) (Figure 1A).^23^

**Figure 1:**
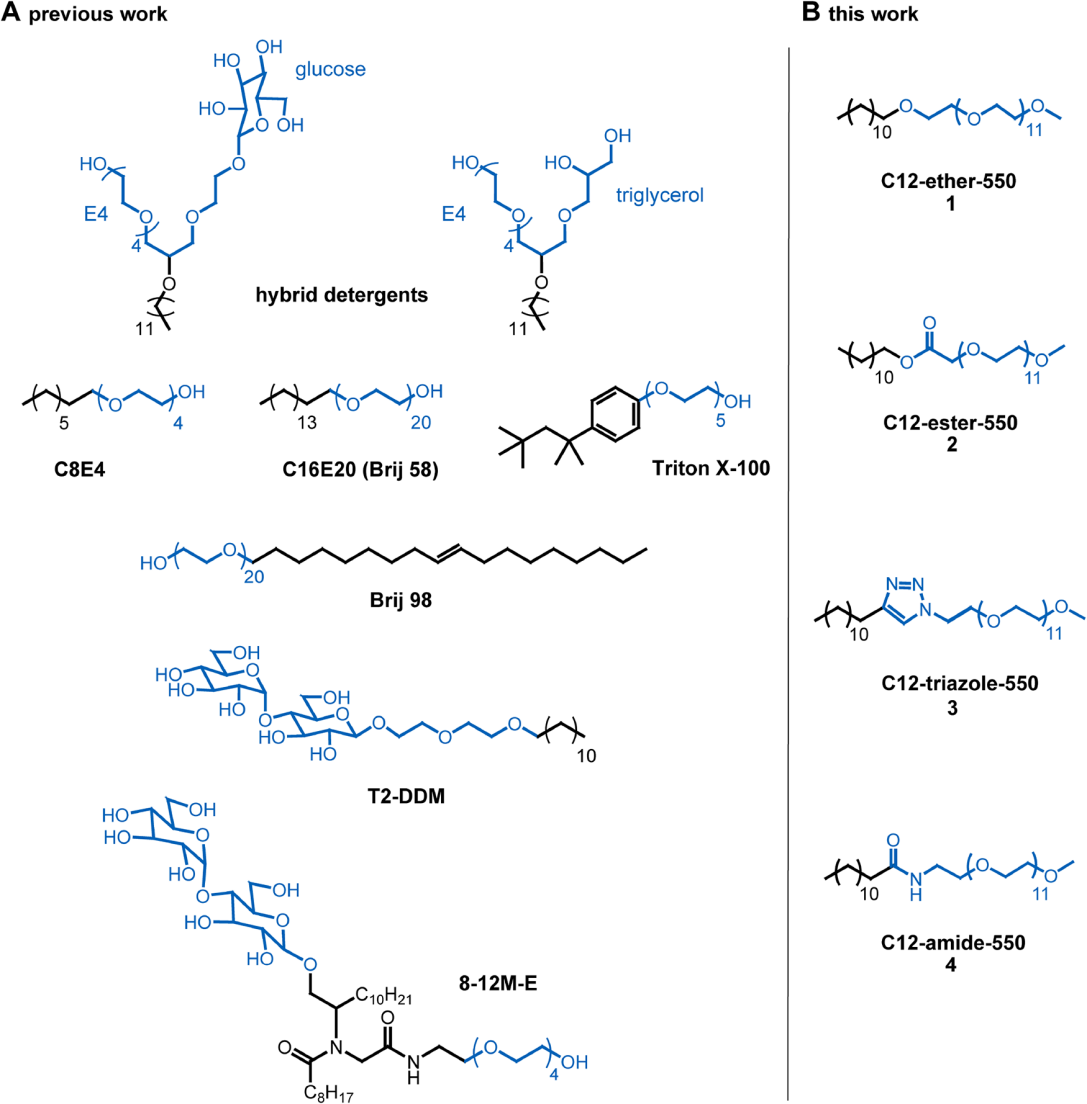
Overview of PEG detergent architectures. (A) PEG detergents currently used in membrane protein research differ in design of the polar head (marked in blue), PEG length or in the design of the nonpolar tail. (B) Molecular structures of PEG550 detergents **1–4** designed and synthesized in this work, which differ in terms of their linkers, such as ether, ester, triazole and amide.

The question left open is whether changing linker polarity can mitigate the denaturing properties that PEG detergents can have on membrane proteins. To address this question, we investigate a series of four PEG550 detergents, namely, C12-ether-550 (**1**), C12-ester-550 (**2**), C12-triazole-550 (**3**), C12-amide-550 (**4**), which differ systematically in the polarity of their linkers (Figure 1B). To enable membrane protein purification trials, we investigated their aggregation behavior with dynamic light scattering (DLS) and confirmed relative differences in the polarity of the linkers with reversed-phase high-performance liquid chromatography (RP-HPLC). The detergents were then analyzed for their ability to purify the vitamin B12 tranporter (BtuCD) from bacterial membranes.

**Scheme 1:**
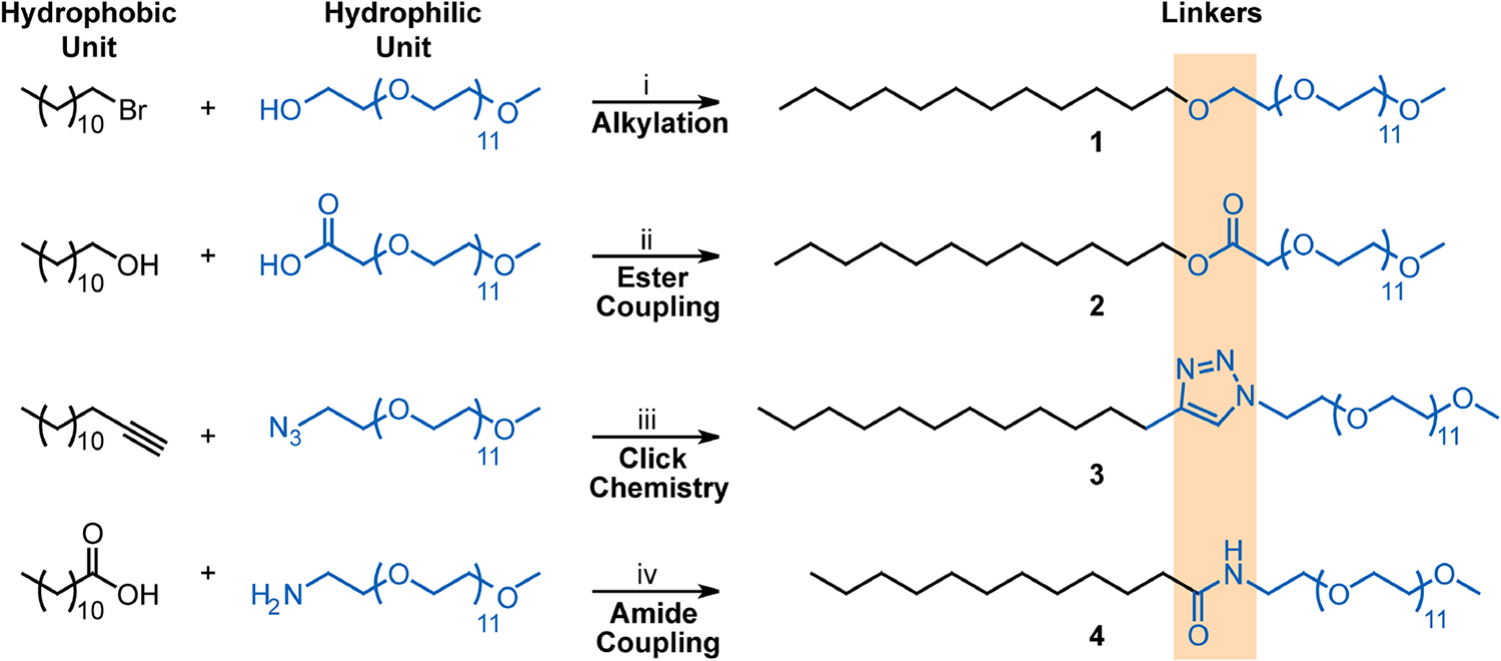
Utilized reaction conditions for the synthesis of PEG550 detergents: (i) NaH, THF, 0-50 °C, 24 h, 67%; (ii) EDC-HCl, DMAP, DMF, 24 h, 61%, 50 °C; (iii) copper sulfate, sodium scorbate, THF/water (3:1, v/v), 50 °C, 24 h, 92%; (iv) EDC-HCl, HOBt, DMF, 60 °C, 24 h, 65%

## Results and Discussion

### Detergent Synthesis

Detergent molecules are known for their structural versatility, which is key to their effectiveness in purifying membrane proteins. Precise synthetic tuning is required to achieve targeted functionality. In this study, we developed a library of amphiphilic detergents consisting of a C12 alkyl chain and commercially available monomethoxy poly­(ethylene glycol) (PEG550). This headgroup is an attractive starting material for detergent synthesis. It is commercially available, cheap, water-soluble, and contains an independently addressable hydroxyl group.^24^ To gradually change the polarity of the linker between head and tail, PEG550 was connected to hydrophobic tails by four different synthetic strategies, i.e., O-alkylation, ester coupling, click chemistry, and amide coupling. Alkylation of PEG550 with 1-bromododecane gave the ether-linked derivative **1** (Scheme 1). To obtain the carboxylic acid-functionalized monomer, PEG550 was oxidized with potassium permanganate (KMnO_4_) under basic conditions.^25^ The ester-linked detergent was synthesized by Steglich esterification between PEG550-COOH and dodecanol (C12-OH) **2** (Scheme 1). Subsequently, azide-functionalized PEG550-N_3_ was prepared in a two-step procedure by mesylation of the hydroxyl group in PEG550 and subsequent nucleophilic substitution with sodium azide. Subsequent catalytic hydrogenation with Pd/C under hydrogen atmosphere reduced the azide to the corresponding primary amine to obtain PEG550-NH_2_.^26^ To obtain the triazole-linked detergent **3**, the azide-functionalized PEG550 was coupled via a copper-catalyzed azide–alkyne cycloaddition with a terminal alkyne-bearing C12 (Scheme 1). Finally, to obtain the amide-linked detergent **4**, PEG550-NH_2_ was coupled with dodecanoic acid (C12-COOH) using standard amide coupling conditions (Scheme 1). The polymeric nature of the commercially available, monofunctional PEG550 ^27^ posed an analytical challenge in detergent characterization. The polydispersity and heterogeneity of PEG550 led to detergent mixtures. We obtained fingerprint mass spectra showing the distribution of PEG550 detergents that differ in the lengths of their oligoethylene glycol repetition units, including species differing by masses of 16 and 28 Da, which is typical for PEG-based materials and arises from oxygen- and carbon-related fragmentation pathways during ionization (Figures S1–S4).^28,29^ We confirmed product formation by alternative measures with thin-layer chromatography and nuclear magnetic resonance spectroscopy, including the assignment of signals related to the characteristic functional groups obtained upon detergent synthesis. Our findings spotlight the challenge of using ill-defined polymeric materials for the preparation of small molecules, warranting further investigations regarding identification and quantification of PEG550 and related materials in the future.^30^ For didactic purposes, we proceeded with our discussion by referring to the representative molecular structures shown in Figure 1 and Scheme 1.

### Physicochemical Studies

The critical aggregation concentration (cac) is the concentration at which detergents begin to form aggregates. The cac value is the minimum information required from a new detergent to do protein purification tests.^31,32^ To ensure detergents can solubilize hydrophobic membrane proteins by shielding hydrophobic surfaces from water, detergent concentrations in purification tests are typically adjusted above the cac.^33^ To investigate whether changing the linker of PEG550 detergents affects cac values, we employed fluorescence spectroscopy to monitor the detergent-concentration-dependent solubilization of a dye.^34^ All synthesized PEG550 detergents exhibited comparable cac values, ranging from 0.45 to 0.5 mg/mL, indicating that changing the polarity of the linker had a minimal effect on the cac (Figures 2A and S5). In addition, the aggregation behavior of the detergents was evaluated using DLS. The volume distribution profiles indicate the formation of small aggregates with hydrodynamic diameters between 3 to 4 nm (Figure 2B), which is similar to the aggregation behavior obtained from micelle-forming PEG detergents.^9,35^ Altogether, these results suggest that particles formed by PEG550 detergents **1–4** in solution above cac have hydrodynamic diameters that are frequently observed in the cases of micelle-forming detergents.^10^

**Figure 2:**
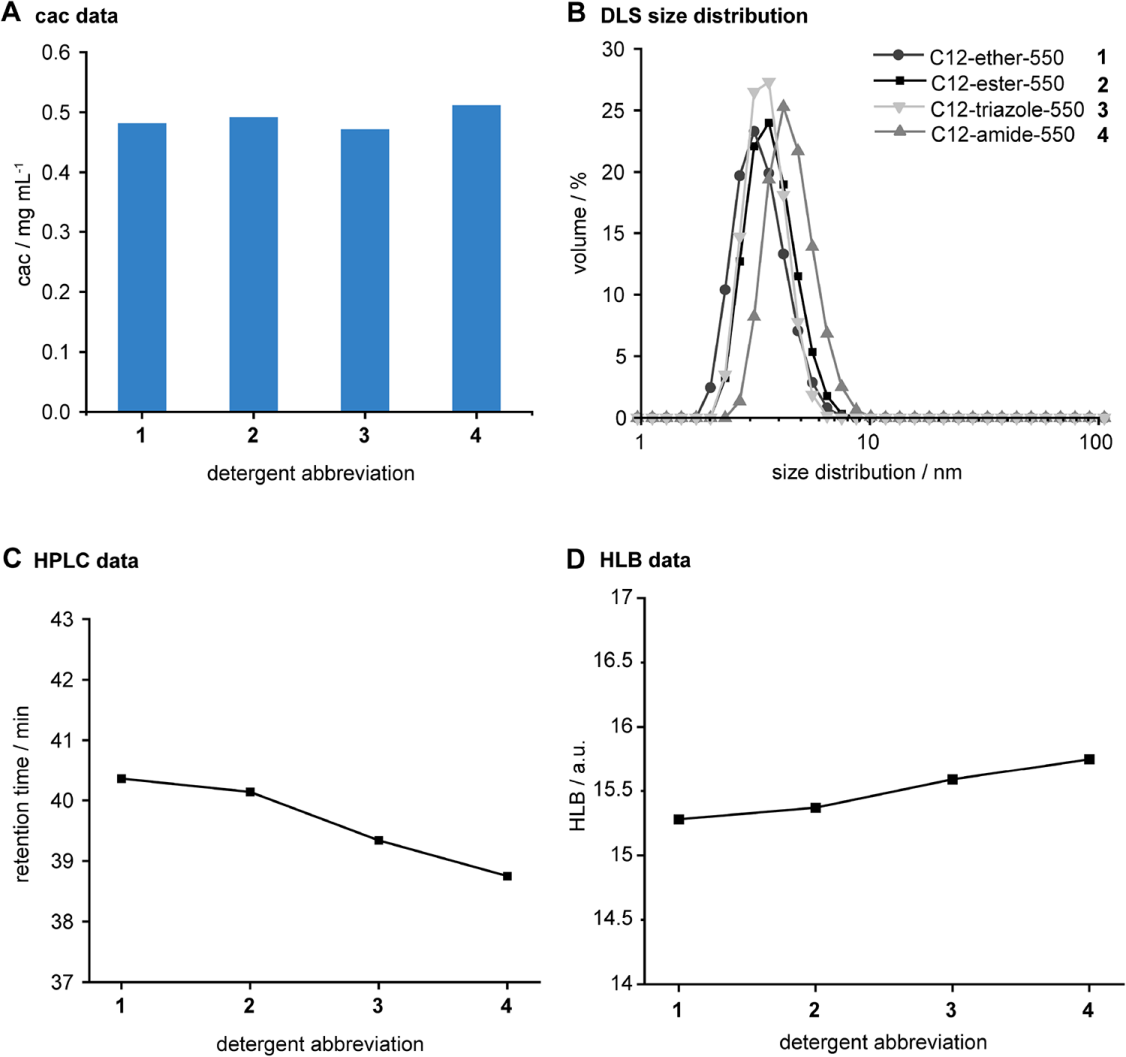
Physicochemical characterization of PEG550 detergents. (A) Bar chart showing cac values determined by using Nile Red as probe plotted against the detergent abbreviations. (B) DLS data showing the volume percent of the size distribution of detergent particles in solution. (C) HPLC data and trendline with retention time plotted against detergent abbreviation. (D) HLB data and trendline with HLB values are shown for different detergent abbreviations.

The cac is sensitive to the overall hydrophobicity of detergents. Since the PEG550 detergents **1–4** had similar cac values, we asked whether changing the linker in PEG550 detergents can noticeably affect their polarity. To compare the overall polarity of our PEG550 detergents, we leveraged a recently established reversed-phase HPLC method and compared their retention times under comparable isocratic elution conditions.^10^ The retention times of our PEG550 detergents decreased from **1** to **4**, suggesting that detergent polarity increased from ether < ester < triazole < amide (Figure 2C). These results correlate well with the respective hydrophilic–lipophilic balance (HLB) values, suggesting that the polarity of the linker between head and tail in PEG550 detergents can be gradually increased by changing the linker in the direction from **1** to **4** (Figure 2C,D). Even though changing the linker in our PEG550 detergent can noticeably affect their overall polarity, we observed similar cac values. The cac of PEG550 detergents is mainly determined by the balance between amphiphilic headgroups and nonpolar tails under the employed experimental conditions.

### Membrane Protein Purification

To assess how changing the polarity of the linkers in PEG550 detergents **1–4** affects the purification of intact membrane proteins, we designed an experimental pipeline that includes standard purification techniques, including overexpression of His-tagged BtuCD in*Escherichia coli*, membrane extraction, affinity purification, and detergent exchange (Figure 3A). First, BtuCD was extracted from membranes with detergents. Second, the protein was purified by immobilized metal affinity chromatography (IMAC) and relative protein quantities were monitored by UV/vis spectroscopy. Third, relative protein quantities were compared to n-dodecyl-β-d-maltoside (DDM), which is a gold standard that delivers high relative protein quantities under the employed conditions.^36^ As a model protein, we chose BtuCD as it belongs to the medically relevant class of ABC transporters that served as positive control in our study, because it has been purified by a range of detergents before under the experimental conditions employed.^37–40^

**Figure 3:**
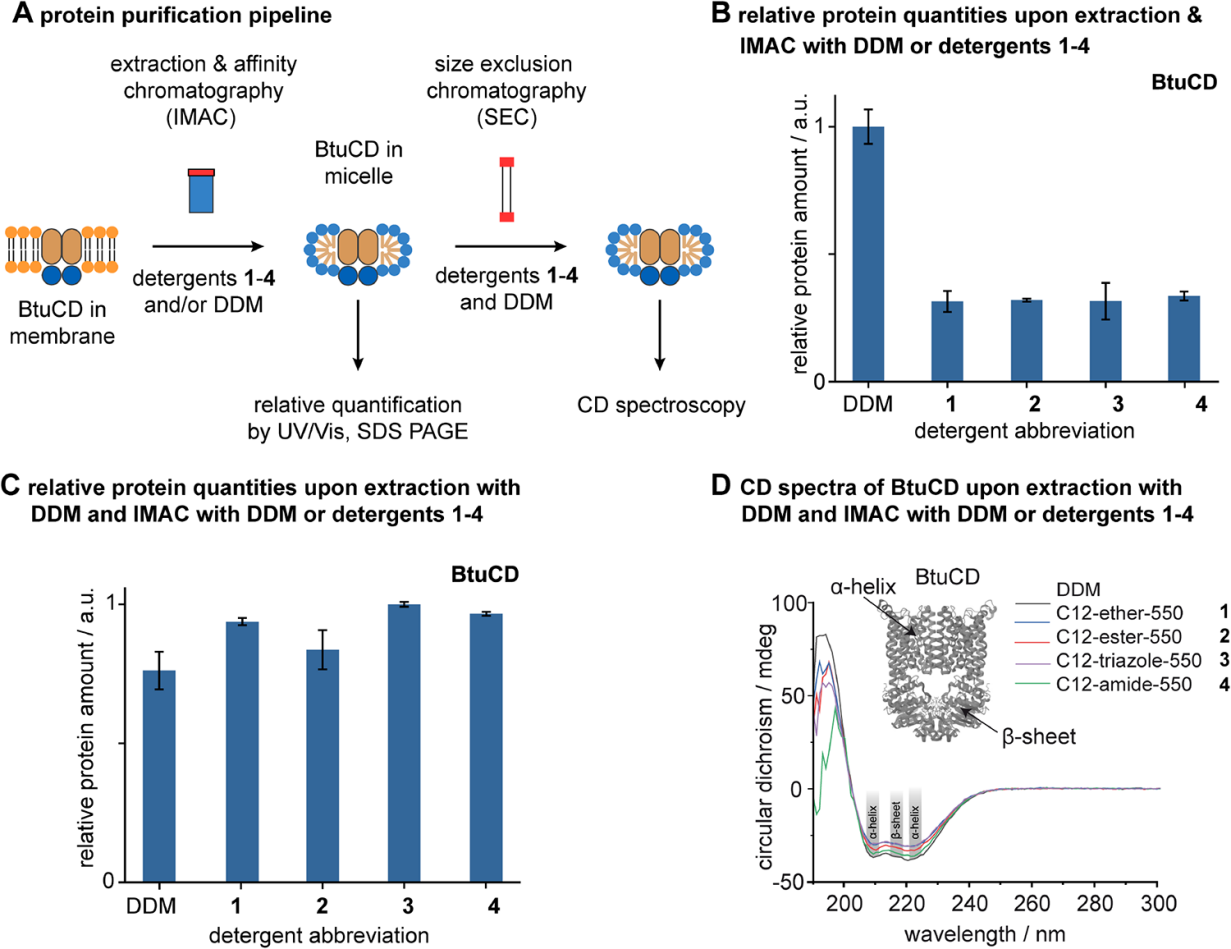
Experimental membrane protein purification pipeline. (A) Schematic visualizing the individual steps of our protein purification pipeline. (B) Bar chart showing the relative protein amount obtained after extraction and IMAC against the detergent abbreviation. (C) Bar chart showing the relative protein amount obtained after extraction with DDM and IMAC against the detergent abbreviation. (D) CD spectra of BtuCD obtained upon extraction with DDM and IMAC with DDM or detergents **1–4**. Crystal structure of BtuCD was adapted with permission from Locher, K. P.; Lee, A. T.; Rees, D. C. The*E. coli* BtuCD structure: a framework for ABC transporter architecture and mechanism. *Science* **2002**, 296, 1091–1098. Copyright 2002, The American Association for the Advancement of Science.

To assess the general utility of **1–4** for protein purification, we compared relative protein amounts obtained upon extraction and IMAC with DDM (Figure 3B). Lower relative protein amounts were consistently obtained from PEG550 detergents **1–4** compared to DDM. Our results indicate that PEG550 detergents **1–4** are not suitable for the purification of large relative protein amounts through extraction and IMAC. The low relative protein amounts obtained from the PEG550 detergents **1–4** can be explained in two ways: the PEG550 detergents did not extract BtuCD from membranes or did not stabilize the protein upon extraction and IMAC. To answer the question of whether our PEG550 detergents can stabilize membrane proteins during IMAC, we extracted BtuCD with DDM and purified the protein during IMAC with DDM or PEG550 detergents **1–4**. We observed comparable relative protein amounts, regardless of the detergent (Figure 3C). This indicates that our PEG550 detergents **1–4** cannot efficiently extract proteins from membranes as good as DDM but are suitable for solubilizing membrane proteins upon detergent exchange from DDM into **1–4** during IMAC.

PEG550 detergents can exhibit more denaturing properties than glycan-based detergents.^1,32^ This led us to the question of whether our PEG550 detergents **1–4** can preserve the secondary structure of BtuCD during extraction and IMAC as good as DDM. To address this question, we analyzed the secondary structure of our BtuCD preparations with CD spectroscopy under comparable conditions. For comparison, a crystal structure of BtuCD is shown whose α-helices and β-sheets are exemplarily indicated with arrows (Figure 3D).^41^ Representative spectral features for α-helices and β-sheets are highlighted accordingly.^42^ The CD spectra obtained from BtuCD in PEG550 detergents **1–4** did not align with the spectrum obtained with DDM (Figure 3D). The overall shapes of the CD spectra were similar, which indicates that the overall fold of BtuCD was partially maintained in our PEG550 detergents **1–4** (Figure 3D). However, since the absolute intensities of the spectra obtained from BtuCD in our PEG550 detergents **1–4** did not match those obtained in the spectrum obtained from DDM, we conclude that BtuCD was partially unfolded. Our PEG550 detergents **1–4** did not preserve the secondary structure of BtuCD upon IMAC as good as DDM.

Detergents find applications in membrane protein research beyond the purification of membrane proteins by extraction, IMAC, and SEC. For example, to test the utility of detergents in maintaining protein stability and functionality^43^ and in native mass spectrometry,^44,45^ membrane proteins are frequently purified in DDM and diluted into detergents whose chemical properties facilitate the acquisition of experimental data. This led us to the question of whether PEG550 detergents can stabilize the secondary structure of BtuCD in aqueous solution following dilution from DDM into **1–4**. To answer this question, we purified BtuCD in DDM, diluted the protein 1:10 (v/v) into buffer containing our PEG550 detergents **1–4**, and measured CD spectra of BtuCD (Figure 4A).

**Figure 4:**
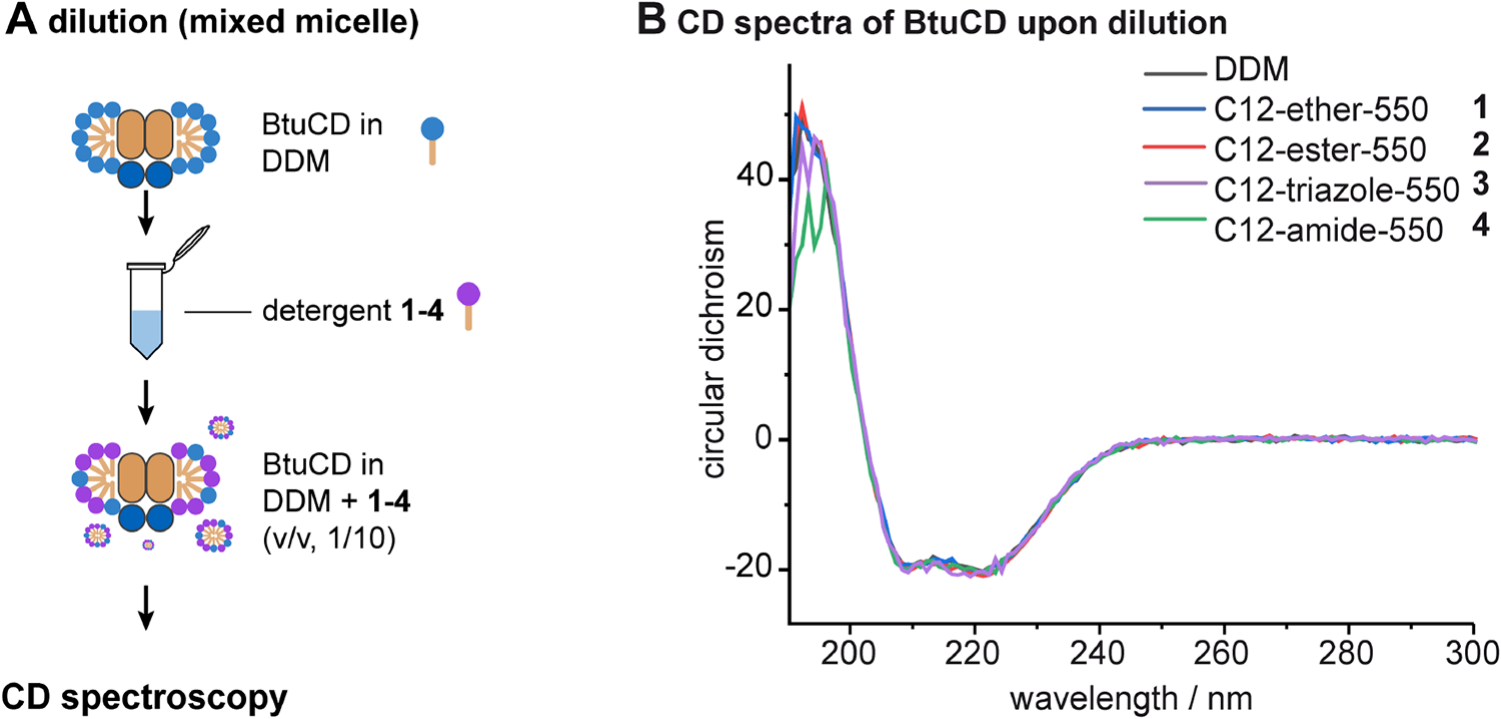
CD spectroscopy following detergent exchange with drop dilution. (A) Schematic showing the individual steps of the drop dilution experiment. (B) CD spectra of BtuCD obtained upon purification, extraction and IMAC with DDM and dilution into DDM or PEG550 detergents **1–4**.

The overall shapes and absolute intensities of the CD spectra of BtuCD obtained upon dilution into our PEG550 detergents **1–4** perfectly matched the reference spectrum taken from BtuCD in DDM (Figure 4B). This led us to the conclusion that our PEG550 detergents **1–4** preserved the secondary structure of BtuCD as good as DDM upon dilution, regardless of the linker between head and tail. In summary, our experiments indicate that the ability of PEG550 detergents **1–4** to solubilize and stabilize the secondary structure of membrane proteins depends on both the structure of the detergent and the way detergent and protein are brought together. We speculated, in the case of the dilution experiment, that the stabilization of the secondary structure of BtuCD may be secured by the residual amounts of DDM that were left within the samples, i.e., 19.3 μg/mL (Table S2).

To test whether adding such residual amounts of DDM to denaturing detergents at 2× cac be used as a strategy to mitigate protein denaturation during extraction and affinity purification, we conducted a detergent screening on BtuCD with buffer containing detergent **1** at 2× cac in the presence and absence of 19.3 μg/mL DDM. The particle size distributions obtained by DLS from both detergent samples looked similar, suggesting that DDM does not alter the size of the formed aggregates (Figure S8). Upon extraction and affinity purification, similar relative protein amounts were obtained from detergent **1**, regardless of the presence of residual amounts of DDM (Figure S9A). The residual amounts of DDM did not improve relative protein amounts under the employed conditions. Subsequent CD spectroscopy analysis revealed that the CD spectrum obtained from BtuCD purified in **1** with and without residual amounts of DDM did not align with the reference spectrum of BtuCD purified with DDM (Figure S9B). This indicates that adding 19.3 μg/mL of DDM to purification buffers containing denaturing PEG550 detergent at 2× cac cannot mitigate membrane protein denaturation during the employed extraction and affinity purification workflow.

## Discussion

Seen from a broader perspective, the linker between the head and tail in nonionic detergents can play a crucial role in membrane protein research. For example, transition linkers have been used to improve the thermal stability of membrane proteins in nonionic glycan detergents.^2^ Changing the polarity of the linker in nonionic linear and dendritic triglycerol detergents can be used to optimize the amount of membrane proteins that is purified from extraction and IMAC.^17^ Changing the basicity of the linker in nonionic detergents can also be used to optimize charge-reducing properties in native mass spectrometry of membrane proteins, as exemplified by oligoglycerol detergents.^16,46^ Regarding the question that motivated this work, i.e., whether tuning the structure of the linker in PEG550 detergents can optimize protein purification outcomes, we can now say that the linkers in PEG550 detergents **1–4** have no measurable influence on their purification performance. We expect the linkers to be too small compared to nonpolar tails and polar heads so that changing the polarity of the linkers from ether to amide in PEG550 detergents did not improve solubilization performance. Our data indicate that our PEG550 detergents did not extract membrane proteins as good as DDM and induced partial protein unfolding when used in substitution for DDM during IMAC or SEC. This is expected for two reasons. First, the HLB values of our PEG550 detergents lay between 15 and 16, which indicate overall polarities that are frequently observed for detergents that are more suitable for solubilizing proteins upon detergent exchange than for extraction and affinity purification.^45,47^ PEG550 detergents are likely too polar to efficiently solubilize membranes for protein extraction.^9^ Second, PEG of a lower molecular weight, e.g., 400 Da, can destabilize the secondary structures of proteins.^48^ Our data complement this knowledge by clarifying that the intrinsic denaturing characteristic of our PEG in the detergents **1–4**, whose PEG headgroups have an average molecular weight in the range of 549–600 Da, cannot be reversed by tuning the chemistry of the linker, i.e., ether, ester, triazole and amide (Figure 2).

Interestingly, our PEG550 detergents could stabilize BtuCD upon dilution from DDM. The underlying procedure is a well-established method for evaluating the utility of detergents in stabilizing membrane proteins.^15,17,49^ Recently, Yang and co-workers observed comparable ligand binding performance to a GPCR when the protein was purified in a newly designed detergent versus diluted from DDM into the newly designed detergent.^17^ This experimental outcome was used to highlight the utility of the sample dilution method for assessing the utility of detergents for maintaining functional characteristics of membrane proteins during extraction and affinity purification. Our data challenge this view by clarifying that it can make a difference in which way detergent and membrane protein are combined. The drop dilution method alone is not sufficient to benchmark the utility of detergents for the purification and analysis of intact membrane proteins. Control experiments including extraction, affinity purification, and size-exclusion are needed to assess the potential of detergents for the purification of intact membrane proteins.

Furthermore, our nonionic PEG550 detergents were able to stabilize BtuCD for at least 24 h after the protein was diluted from DDM-containing buffer in buffer containing PEG detergent **1–4**. As shown for the PEG550 detergent **1**, diluting BtuCD from DDM into **1** at 2× cac produced a monomodal size-exclusion profile that looked similar to a control sample that was prepared exclusively in DDM (Figure S10). Additionally, the ATPase activity remained unchanged when BtuCD was diluted from DDM into **1–4** at 2× cac compared to BtuCD exclusively purified in DDM (Figure S11). Our PEG550 detergents represent new starting points for structural investigations in which saccharide detergents are used for the purification of intact membrane proteins and exchanged for other detergents that exhibit more favorable physicochemical properties for biophysical measurements. Conceivable applications that could benefit from a dilution of proteins that were purified with saccharide detergents into PEG550 detergents include native mass spectrometry,^44^ cryo-electron microscopy,^50^ and X-ray crystallography of membrane protein complexes.^51,52^

## Conclusions

In summary, we synthesized four nonionic PEG550 detergents containing different linkers, namely ethers, esters, triazoles and amides. We investigated their physicochemical properties, including cac, polarity, and hydrodynamic radii of aggregates formed above cac, which enabled us to employ these detergents in membrane protein purification. We found that PEG550 detergents **1–4** could not extract the ABC transporter BtuCD from lysed bacterial membranes, regardless of the linker. Furthermore, CD spectroscopy revealed that PEG550 detergents **1–4** could not stabilize the secondary structure of BtuCD after IMAC as good as DDM. In contrast, after purification of BtuCD with DDM and dilution into CD spectroscopy buffer containing PEG550 detergents it was possible to maintain the secondary structure, monodispersity, and ATPase activity of BtuCD as good as with DDM. Changing the linker in PEG550 detergents did not improve relative protein amounts upon extraction and IMAC, including the retention of secondary structure. The chemistry of the linker did not induce protein denaturation upon drop dilution. The denaturing properties of PEG550 detergents depend more likely on the amphiphilic properties of the PEG550 headgroup and can be controlled by the technique with which membrane protein and detergent are brought together, i.e., extraction and affinity purification or detergent exchange. Headgroup structure and detergent exchange determine the utility of PEG550 detergents for membrane protein purification. This conclusion was derived from investigating one subclass of PEG550 detergents and BtuCD. Future exploration is required to evaluate the validity of this conclusion for the broader chemical space of PEG detergents. In this regard it would be interesting to proceed with the in-depth characterization and quantification of PEG550, including related materials, and testing transferability of our findings to PEG detergents with free terminal hydroxyl groups, as found in most conventional PEG detergents. In general, the variety of detergents has been expanded over the past decades.^53^ The number of new PEG detergents entering the field has seen a modest increase compared to glycan or oligoglycerol detergents.^20^ Our findings deliver a new starting point for the streamlined optimization of sample properties with PEG550 detergents for potential applications in biophysical measurements with membrane proteins.

## Experimental Section

### Materials and Method

All chemicals and solvents used were purchased from Sigma-Aldrich Chemicals. Silica gel (60–120 mesh) was used for column chromatography. Deionized water was used to prepare samples for physicochemical characterization and transport studies.

### Critical Aggregation Concentration

The critical aggregation concentration of the synthesized amphiphiles was investigated using the fluorescence measurement technique using “Nile Red” as a model dye. A stock solution of the dye was prepared in THF at a concentration of 1 mg/mL. To form a thin layer, 10 μL of the stock solution was added to each empty vial and then the THF was completely evaporated. The stock solutions of amphiphiles (2.5 mg/mL) were prepared in deionized water. To obtain different concentrations of the amphiphiles, the stock solutions were serially diluted twice and the solution was then transferred to the vial containing the thin dye film and stirred overnight. Poly­(tetrafluoroethylene) (PTFE) filters (0.45 μm) were used to remove the unencapsulated dye from the solutions, and then fluorescence measurements were performed using a Cary Eclipse fluorescence spectrophotometer. To calculate the cac value, the fluorescence intensity was plotted against the logarithm of the detergent concentration. The intersection between baseline and increase in signal intensity was extrapolated and taken as cac.

### Dynamic Light Scattering

The Malvern Zeta Sizer Nano ZS analyzer, integrated with a 4 mW He–Ne laser, λ = 633 nm, using backscattering detection (scattering angle θ = 173°) with an avalanche photodiode detector, was used to determine the size of nanostructures (micelles/aggregates) formed by the supramolecular organization of amphiphiles in deionized water at a concentration of 2.5 mg/mL. The samples were then further mixed for 20 h at 25 °C with vigorous stirring. The resulting solutions were then filtered through a 0.45 μm PTFE filter and allowed to equilibrate for 1 h at room temperature. They were then transferred to disposable ultraviolet (UV) cuvettes from microBRAND and analyzed by DLS.

### HPLC Measurements

Analytical HPLC measurements were performed using a reverse-phase HPLC system from Knauer, equipped with a UV detector and a C18 column (250 × 4.6 mm, 5 μm particle size). A binary mobile phase composed of water and methanol was employed under isocratic conditions. The flow rate was set to 1.0 mL/min, and the column temperature was maintained at 25 °C. An injection volume of 20 μL was used for all analyses with a detergent concentration of 5 mg/mL. All samples were filtered through a 0.45 μm syringe filter prior to injection.

### Calculating HLB Values

The HLB values were calculated according to GRIFFIN’s equation^54^

1 $$\mathrm{HLB}=20\cdot \frac{MW_{\mathrm{tail}}}{MW_{\mathrm{tot}}}$$

with MW_tot_ = molecular weight of the entire molecule and MW_tail_ = molecular weight of the nonpolar tail. The molecular weights of the molecules and tails used for HLB calculations are summarized in Table S1.

### Protein Expression

Plasmids encoding the protein of interest and related antibiotic resistance (ampicillin for His-tagged BtuCD) were transformed by mixing 1 μL of a plasmid-containing solution (100 ng/μL) with a 50 μL aliquot of BL21­(DE3) competent cells. The mixture was incubated on ice for 30 min. The mixture was placed into a water bath with a temperature of 37 °C for 30 s and then the mixture was placed into ice for 90 s. Subsequently, Roth LB Broth Luria/Miller solution (450 μL of a 25 g/L solution) was added, and the mixture was incubated for 30–45 min with 200 rpm at 37 °C. The cells were then plated on agar plates (25 g/L Roth LB Broth Luria/Miller, 15 g/L agar–agar, 50 μg/mL ampicillin) and incubated overnight at 37 °C. Three colonies were picked and transferred into Roth TB-Broth (200 mL of a 2.5 g/L solution, 50 μg/mL ampicillin). The starter culture was incubated with 160 rpm at 37 °C for 8 h. The starter culture was transferred into Roth TB-Broth (12 L of a 2.5 g/L solution, 50 μg/mL ampicillin) and incubated for 16 h with 160 rpm at 28 °C. Subsequently, Isopropyl-β-d-thiogalactopyranosid was added (12 mL of a 1.54 M solution). The cells were shaken with 160 rpm at 37 °C for 4 h. The cells were then harvested by centrifugation (5000 *g*, 10 min, 22 °C) with an Avanti JXN-26 centrifuge (Beckmann Coulter). The supernatant was discarded, and the pellets were collected and stored at −80 °C.

### Membrane Preparation for Protein Purification

The cell pellet obtained after protein expression was suspended in 150 mL buffer (20 mM Tris, 300 mM NaCl, 20v% v/v glycerol, 2 protease inhibitors complete by Roche, pH = 8) and lysed using Avestin Emulsiflex-C5 (900–1500 bar, 4 °C). The supernatant was clarified by centrifugation (20,000 *g*, 20 min, 4 °C). The supernatant was isolated and subjected to centrifugation (100,000 *g*, 1 h, 4 °C) with an Optima XPN 80 Ultracentrifuge (Beckmann Coulter) to obtain a membrane pellet. The supernatant was discarded, and the membrane pellet was homogenized in 9 mL buffer (20 mM Tris, 100 mM NaCl, 20% v/v glycerol, 1 protease inhibitor complete by Roche per 50 mL buffer, pH = 8). The protein-containing membrane suspension was flash frozen and stored at −80 °C.

### Purification of BtuCD by IMAC

All steps of the protein purification procedure were conducted on ice. First, extraction buffer (700 μL of 200 mM NaCl, 20 mM Tris, pH = 8) was mixed with protein-containing membrane suspension (200 μL) and detergent stock (100 μL of a 10% w/v aqueous solution). The mixture was inverted and stored on ice for 2 min. The supernatant was separated by centrifugation (10,000 rpm, 10 min, 4 °C). To purify BtuCD, IMAC was performed. A spin column (Bio-Spin by Bio-Rad) was filled with Ni-NTA resin (800 μL of a 50 w% Ni-NTA agarose suspension, Qiagen), which gives a column volume (CV) of approximately 400 μL. The column was washed with deionized water (1 mL) and loading buffer (1 CV of 200 mM NaCl, 20 mM Tris, 20 mM imidazole, detergent at 2× cac, pH = 8). The protein-containing supernatant (800 μL) was loaded onto the column. The column was washed with detergent-containing loading buffer (3 CVs) and wash buffer (6 CVs of 200 mM NaCl, 20 mM Tris, 40 mM imidazole, detergent at 2× cac, pH = 8). BtuCD was eluted with elution buffer (500 μL of 200 mM NaCl, 20 mM Tris, 200 mM imidazole, detergent at 2× cac, pH = 8). The protein sample (130 μL) was pipetted onto desalting spin columns (Thermo Scientific Zeba Spin Desalting Columns, 7K MWCO) to remove the imidazole by exchanging the elution buffer against extraction buffer containing the detergent of interest at 2× cac. The elution volumes of the protein samples were determined with an Eppendorf pipet. The sample absorption at 280 nm was recorded by means of an Implen NanoPhotometer NP80. The relative protein amounts obtained upon IMAC were visualized by plotting the absorption at 280 nm (A280) against the tested detergents. The average A280 values from two independent repeats (*n* = 2) were determined, including the standard error of the mean (SEM). The average A280 values (±SEM) were normalized to the largest value obtained among the investigated detergents and plotted against the tested detergents. Subsequently, the volume-corrected absorption values were determined, normalized and plotted against the tested detergents. For details on the volume correction of absorption values, please see ref 33.

### SDS-PAGE Gel Electrophoresis

To evaluate relative purity of the protein preparation, an aliquot of the protein solution (4 μL) obtained upon IMAC was mixed with an aliquot of ROTILoad 1 buffer by Roth (4 μL) and loaded onto a SDS Page gel (Mini-PROTEAN TGX Stain-Free Gels by Bio-Rad). Gel electrophoresis was conducted (200 V, 30 min) and the gel image was visualized and recorded by means of ChemiDoc MP Imaging System by Bio-Rad (Stain free gel, UV transillumination, 138 s activation time, 5081 s exposure time (optimal Autoexposure)) (Figure S7).

### CD Spectroscopy

Protein solutions obtained upon IMAC were transferred into CD spectroscopy buffer (using desalting columns (CV = 5 mL, Cytiva HiTrap Desalting, product number: GE17–1408–01)). Columns were washed with deionized water (3 CVs) and equilibrated with detergent-containing CD spectroscopy buffer (2 CVs of 100 mM (NH_4_)­HCO_3_, pH = 8, detergent of interest at 2× cac). Protein solutions obtained upon IMAC (∼0.5 mL) were injected manually into the columns using syringes. The proteins were eluted with CD spectroscopy buffer (2 CVs of 100 mM (NH_4_)­HCO_3_, pH = 8, detergent of interest at 2× cac) and fractions were collected (fraction size = 1 mL). Protein-containing fractions were identified by UV spectroscopy, combined, and concentrated to a final protein concentration of 2.78 μM. So-obtained protein solutions were loaded into cuvettes (Hellma Analytics, Quartz Suprasil, volume = 350 μL, layer thickness = 1 mm, product number: HL110–1–40). The CD spectrometer (Chirascan Applied Photophysics qCD) was purged with nitrogen for 1 h. The following experimental parameters were used: temperature (4 °C), wavelength range (190–300 nm), step size (1 nm), scan speed (0.5 s/point), bandwidth (0.5 nm), and repeats per sample (3). The average CD intensity of three scans was plotted against the wavelength. Detergent-containing CD spectroscopy buffers were used as blanks. Data were acquired with Pro-Data Chirascan V4.5 and analyzed with Origin V10.2.

### CD Spectroscopy upon Drop Dilution

For preparation of CD spectroscopy samples following detergent exchange by dilution, six BtuCD purifications with DDM were done in parallel as described above. The protein solutions were combined and concentrated with centrifugal filters (Amicon Ultra-15 mL Centrifugal Filters, 100 kDa MWCO by Millipore) to a volume of about 1.5 mL. The concentrated protein solution was transferred into CD spectroscopy buffer (100 mM (NH_4_)­HCO_3_) as described above—see subsection “CD Spectroscopy.” The protein-containing fractions were combined, reduced to a volume of 1 mL with centrifugal filters, and subjected to another round of buffer exchange. The protein containing fraction (1 mL) was centrifuged (10,000 rpm, 4 °C) in a centrifugal filter (Amicon Ultra 0.5 mL Centrifugal Filters, 100 kDa MWCO by Millipore) to a final volume of 300 μL (A280 = 0.3). BtuCD-containing aliquots (44 μL) were diluted into 200 μL of CD spectroscopy buffer (100 mM (NH_4_)­HCO_3_, pH = 8, containing the detergent of interest at 2× cac, e.g., DDM, or **1–4**). The samples were analyzed by CD spectroscopy as described before in subsection “CD spectroscopy.”

### Relative Detergent Quantification

LC-MS experiments were done with a high-pressure liquid chromatography (HPLC) time-of-flight (TOF) mass spectrometry (MS). The device Agilent 1260 Infinity II system (Agilent Technologies, Waldbronn, Germany) was equipped with a G7129A autosampler, a G7116A column oven, a G7117C photodiode array detector and a G7111B quaternary pump system. The measurement was performed on a compact QTOF (Bruker Daltonics GmbH & Co. KG, Bremen, Germany). Mass calibration were carried out using ESI­(+). To investigate whether the calculated concentration of residual DDM after drop dilution corresponded to 19.3 μg/mL, we conducted a relative quantification. We compared the peak intensities of DDM and G1 OGD in the chromatograms obtained from samples containing DDM (19.3 μg/mL) and G1 OGD (220 μg/mL). We repeated this experiment with samples containing G1 OGD (220 μg/mL) and BtuCD that was purified in DDM and diluted into detergent **1** at 2× cac. To confirm the DDM concentration of 19.3 μg/mL in both samples, the ratio of the peak intensities obtained from G1 OGD and DDM in both samples was compared (Table S2).

## Supplementary Material



## Notes

Fingerprint mass spectrometry, cac analysis, DLS analysis of PEG550 detergents **1–4**, SDS-PAGE gels, DLS analysis of detergent mixtures, utility of detergent mixtures for protein purification, SEC elution profiles, ATPase activity assay, HLB values, relative quantification of DDM in drop dilution, synthesis procedures of PEG550 detergent **1–4**, NMR data, experimental procedures of activity assay and SEC elution profile (PDF)
