# Supplementary material for: Drop Dilution Enables the Use of PEG-Derived Detergents for Membrane Protein Purification
Source: ACS Omega. 2025 Nov 14;10(46):56670–9. doi: 10.1021/acsomega.5c09173 (PMC12658807; doi:10.1021/acsomega.5c09173)
Supplement: Supplementary file 1 [file ao5c09173_si_001.pdf]

Supporting Information

Drop Dilution enables the use of PEG-derived Detergents for Membrane Protein Purification

Katharina Alker‡a, Shweta Singh‡b, Arun K. Vinodakrishnan[c], Florian Lindemann[a], Rasmus Linser[a], Ram Singh*b, Abhishek K. Singh*c, Leonhard H. Urner*a

[a] TU Dortmund University, Department of Chemistry and Chemical Biology, Otto-Hahn-Str. 6, 44227 Dortmund, Germany

[b] Department of Applied Chemistry, Delhi Technological University, Delhi110042, India

[c] Freie Universität Berlin, Institute of Chemistry and Biochemistry, Takustr. 3, 19348 Berlin, Germany

‡ Both authors contributed equally

Email: ramsingh@dtu.ac.in; abhikmc@zedat.fu-berlin.de; leonhard.urner@tu-dortmund.de

Table of contents

Supplementary figures 2

Figure S1: Finger-print mass spectrometry data obtained from C12-Ether-550 1. 2

Figure S2: Finger-print mass spectrometry data obtained from C12-Ester-550 2 3

Figure S3: Finger-print mass spectrometry data obtained from C12-Triazole-550 3. 4

Figure S4: Finger-print mass spectrometry data obtained from C12-Amide-550 4. 5

Figure S5: Cac analysis of PEG550 detergents. 6

Figure S6: DLS analysis by intensity of PEG550 detergents. 7

Figure S7: Gel electrophoresis after purification of BtuCD using detergents 1-4. 8

Figure S8: DLS analysis of detergent mixture. 9

Figure S9: Utility of detergent mixtures for protein purification. 10

Figure S10: SEC elution profiles of BtuCD in DDM and detergent mixture 11

Figure S11: Activity of BtuCD in DDM or PEG550 detergents 1-4 12

Supplementary tables 13

Table S1: HLB values of PEG detergents 1-4. 13

Table S2: Relative quantification of DDM in drop dilution. 14

Supplementary methods 15

Synthetic procedures 15

Synthesis of C12-ether-550 (1) 15

Synthesis of C12-ester-550 (2) 15

Synthesis of C12-triazole-550 (3) 15

Synthesis of C12-amide-550 (4) 16

NMR data of final detergents 17

Experimental procedure Activity Assay 21

Calculation of BtuCD activity 22

SEC elution profile 23

References 24

Supplementary figures

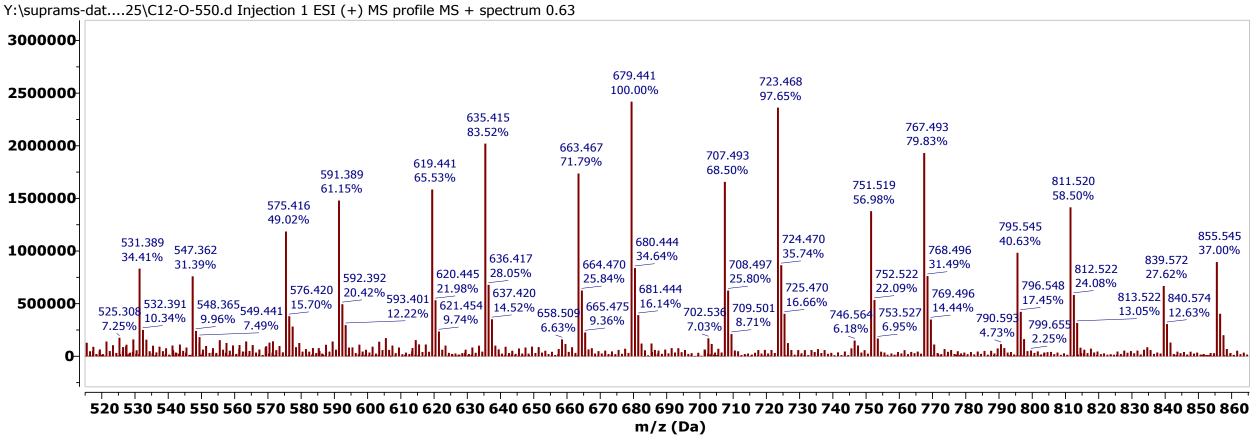

Figure S1: Finger-print mass spectrometry data obtained from C12-Ether-550 1.

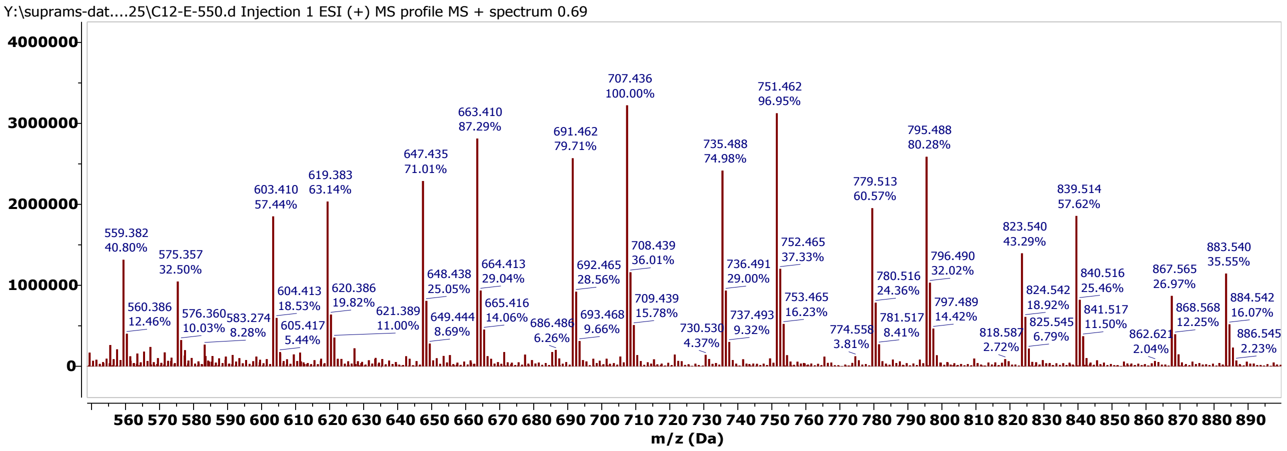

Figure S2: Finger-print mass spectrometry data obtained from C12-Ester-550 2.

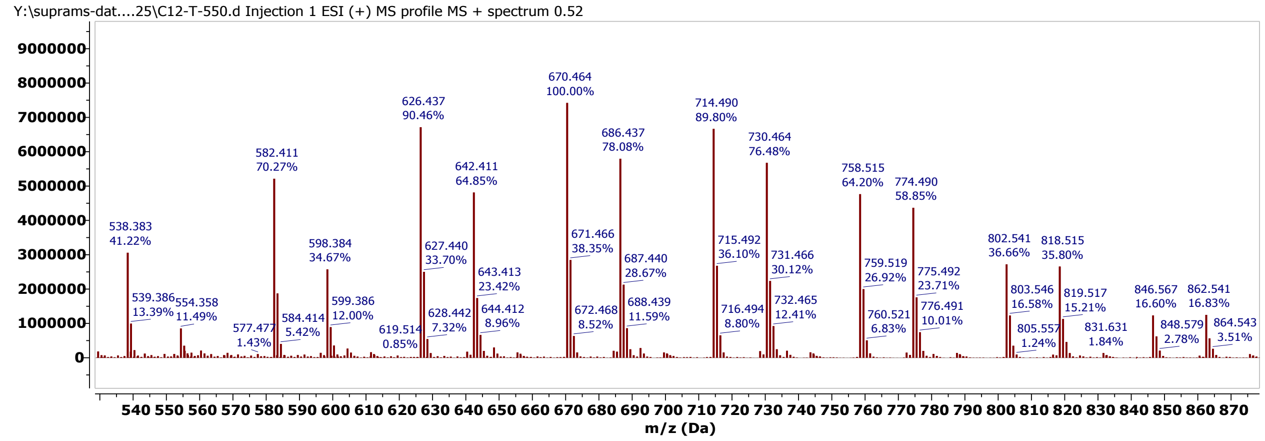

Figure S3: Finger-print mass spectrometry data obtained from C12-Triazole-550 3.

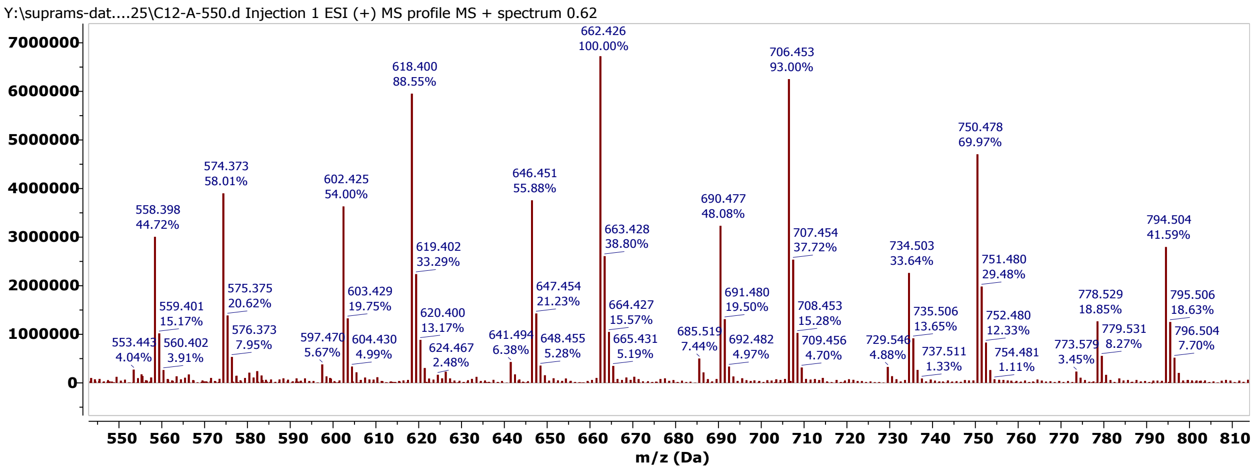

Figure S4: Finger-print mass spectrometry data obtained from C12-Amide-550 4.

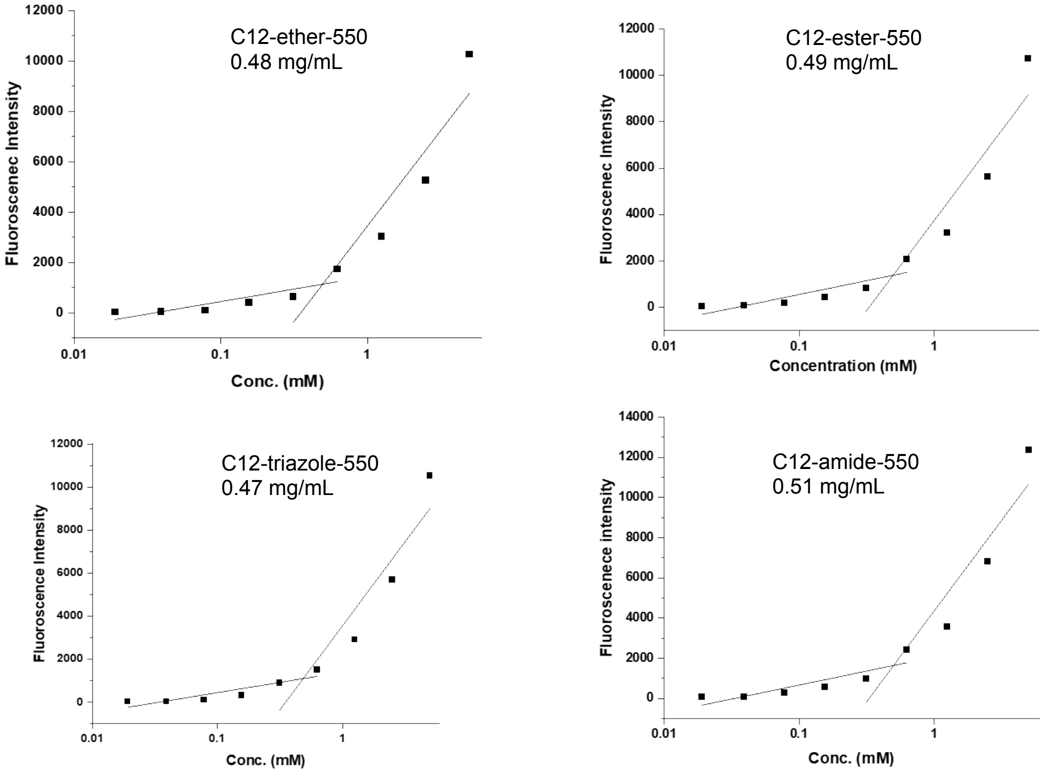

Figure S5: Cac analysis of PEG550 detergents. Determination of the critical micelle concentration of amphiphiles using fluorescence spectroscopy using Nile Red as a probe. The Amphiphiles concentration was 5mg/ml.

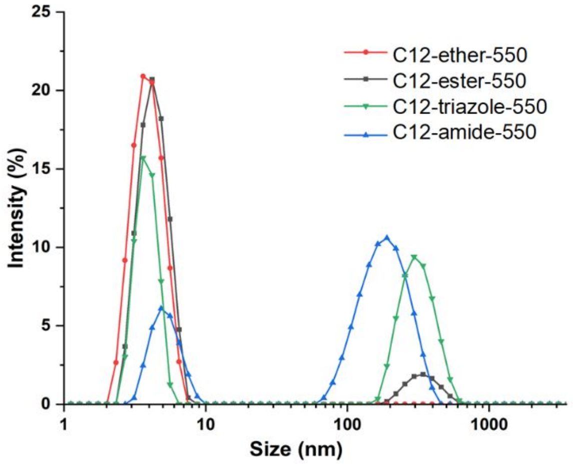

Figure S6: DLS analysis by intensity of PEG550 detergents. Size distribution profile of particles in solution formed by the PEG550 detergents in water above cac.

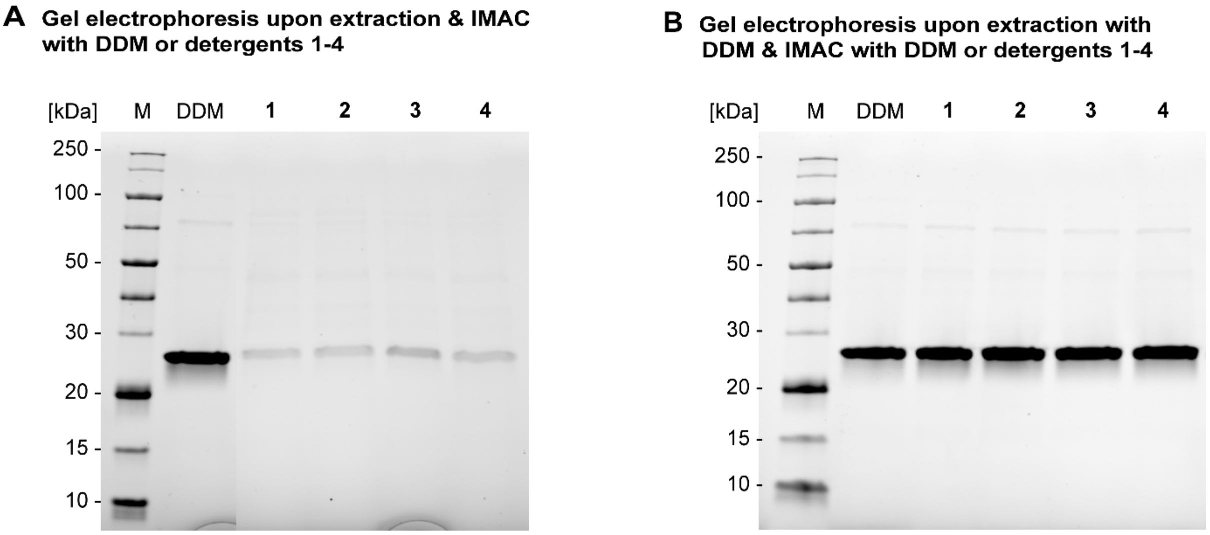

Figure S7: Gel electrophoresis after purification of BtuCD using detergents 1-4. A) SDS PAGE image taken after extraction and IMAC of BtuCD with DDM or detergents **1-4**. B) SDS PAGE image taken after extraction of BtuCD with DDM and IMAC with DDM or detergents **1-4**.

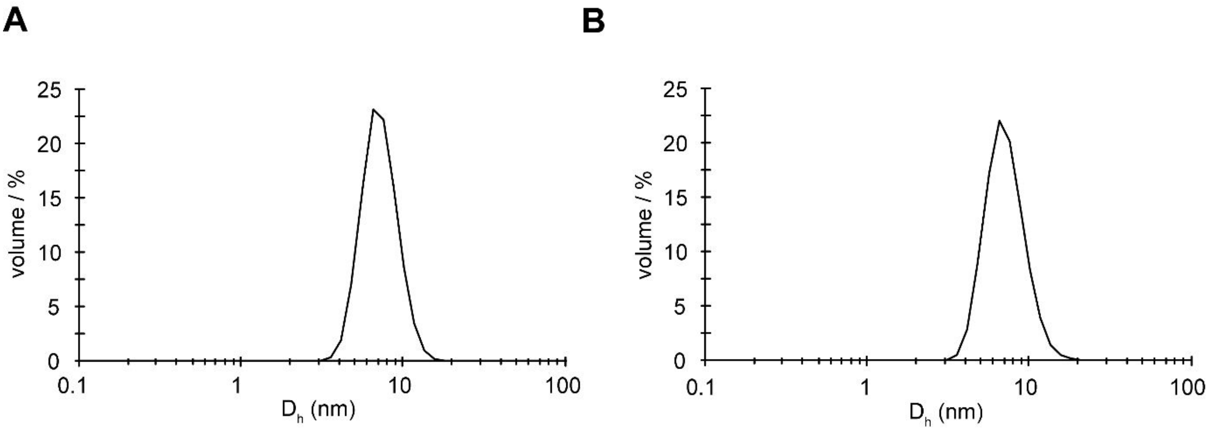

Figure S8: DLS analysis of detergent mixture. Diagram showing diffusion coefficients of particle size distributions by volume % of A) C12-ether-550 (2x cac) and B) C12-ether-550 (2x cac) + 19.3 µg/mL DDM.

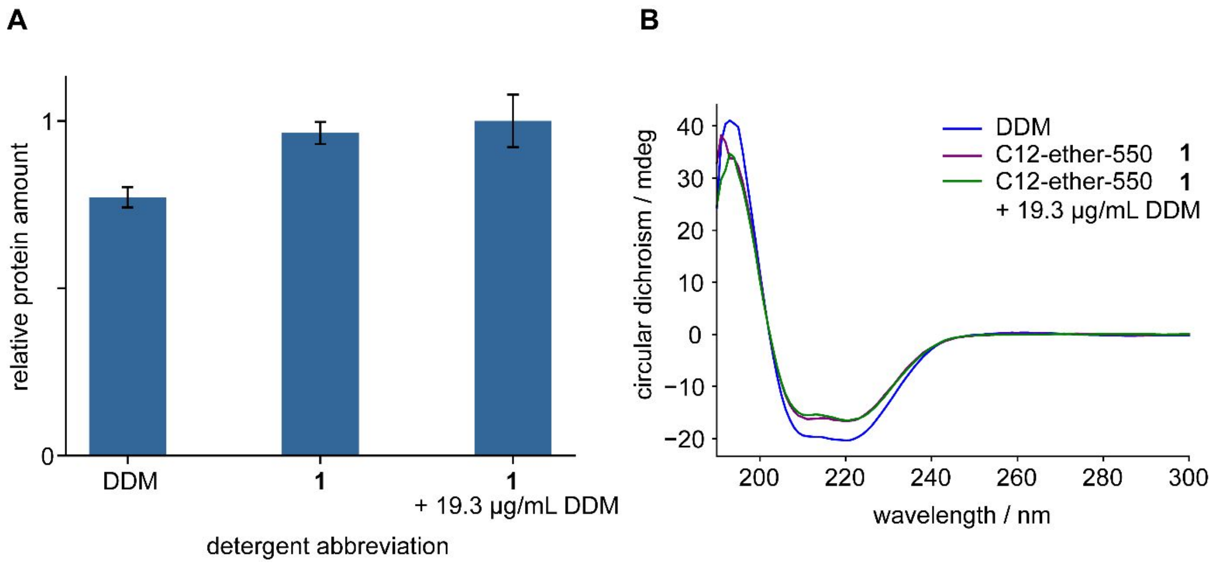

Figure S9: Utility of detergent mixtures for protein purification. A) Bar chart showing relative protein amounts of purified BtuCD against DDM, detergent **1** or detergent **1** + 19.3µg/mL DDM. **B)** Diagram showing circular dichroism spectra of purified BtuCD with DDM, detergent **1** or detergent **1** + 19.3 µg/mL DDM.

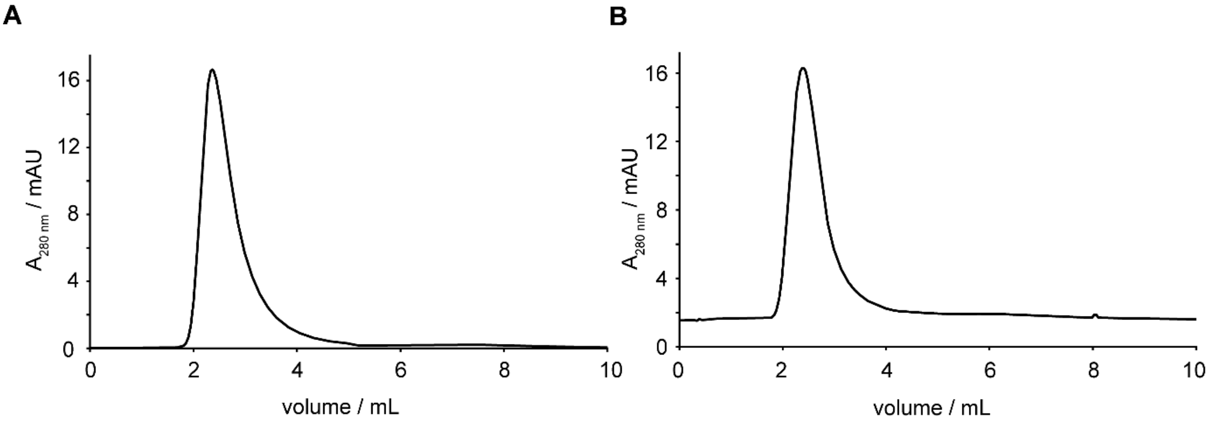

Figure S10: SEC elution profiles of BtuCD in DDM and detergent mixture. Spectogram showing absorbance at 280 nm against the volume of BtuCD containing solution eluted from SEC after A) purification and dilution in DDM and B) purification in DDM and dilution in C12-ether-550.

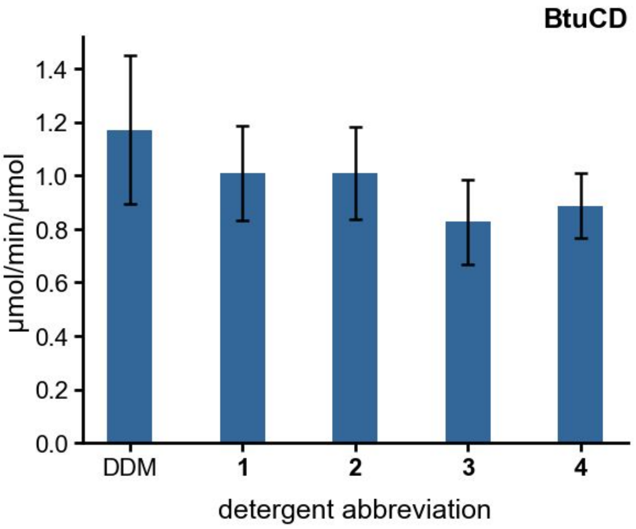

Figure S11: Activity of BtuCD in DDM or PEG550 detergents 1-4. Bar chart showing the activity of DDM purified BtuCD in µmol/min/µmol after dilution in DDM or PEG550 **1-4** detergents.

Supplementary tables

Table S1: HLB values of PEG detergents 1-4. Summary of detergents (detergent), total molecular weight (MW_{tot}), molecular weight of the lipophilic unit (MW_{tail}), molecular weight of the polar unit (MW_{pol}), and hydrophilic-lipophilic balance (HLB).

detergent	MW_{tot}	MW_{tail}	MW_{pol}	HLB
C12-ester-550 (**1**)	718.33	169.33	549	15.28
C12-ether-550 (**2**)	732.33	169.33	563	15.37
C12-triazole-550 (**3**)	769.33	169.33	600	15.59
C12-amide-550 (**4**)	731.18	155.18	576	15.75

Table S2: Relative quantification of DDM in drop dilution. Summary of LC-MS data obtained from DDM (19.3 µg/mL) and G1 (1x cac) (sample 1) and BtuCD purified in DDM and diluted into detergent **1** (v:v, 1:9) + G1 (1x cac) (sample 2), including retention time, observed mass, assigned detergent, calculated mass, ion intensity and intensity ration G1/DDM**.**

sample	retention time [min]	observed mass $[M+Na]^+$	assigned detergent	calculated mass $[M+Na]^+$	ion intensity	intensity ratio G1 OGD/ DDM
**reference**						
**DDM (19.3 µg/mL) + G1 OGD (1x cac)**	18.2	533.29	DDM	533.30	$1.3*10^6$	6.38
	19.8	431.29	G1	431.31	$8.3*10^6$	
**drop dilution**						
**DDM purified BtuCD 1/10 diluted + G1 OGD (1x cac)**	18.3	533.29	DDM	533.30	$1.3*10^6$	6.38
	19.8	431.29	G1	431.31	$8.3*10^6$	

Supplementary methods

Synthetic procedures

Synthesis of C12-ether-550 (1)

Sodium hydride (0.075 g, 1.7 equiv.) was added to a flame-dried round bottom flask, followed by 25 mL of dry tetrahydrofuran (THF). The flask was purged with argon and cooled in an ice bath. In a separate flask, mPEG-OH (1.0 g, 1.0 equiv.) was dissolved in 20 mL of dry THF. This solution was added dropwise to the reaction flask at freezing temperature. The reaction mixture was stirred for 30 minutes at room temperature. Then 1-bromodecane (0.42 g, 1.2 equiv.) was added and the reaction mixture was stirred at 50 °C for 24 hours. The progress of the reaction was monitored by thin layer chromatography (TLC). After completion of the reaction, the solvent was removed under reduced pressure and the crude residue was extracted with dichloromethane (DCM) and water. The aqueous layer was washed twice with DCM (2 × 50 mL), and the combined organic phases were dried over anhydrous sodium sulfate. The solvent was evaporated under reduced pressure, and the crude product was purified by column chromatography using a methanol–DCM gradient as eluent. The yield of the final product was recorded to be 67%.

^{1}H NMR (500 MHz, CD_2Cl_2) δ 3.59 – 3.49 (m, 44H), 3.40 (t, 2H), 3.33 (s, 3H), 1.57-1.51 (m, 2H), 1.37-1.26 (m, 18H), 0.87 (t, 3H). **^{13}C NMR (126 MHz, CD_2Cl_2)** δ 71.89, 71.35, 70.56, 70.50, 70.37, 70.06, 58.63, 31.93, 29.74, 29.68, 29.65, 29.64, 29.52, 29.37, 26.14, 22.70, 13.91ppm.

Synthesis of C12-ester-550 (2)

mPEG-550-COOH (1.0 g, 1.0 equiv.) was dissolved in 40 mL dry DMF in a 100 mL round bottom flask. Dodecanol (0.40 g, 1.2 equiv.) was added, followed by the addition of EDC-HCl (0.42 g, 1.2 equiv.) and DMAP (0.25 g, 1.1 equiv.). The reaction mixture was stirred at 50 °C for 24 hours. The progress of the reaction was monitored by thin layer chromatography (TLC). After completion of the reaction, the DMF was removed under reduced pressure and the crude residue was extracted with dichloromethane (DCM) and water. The aqueous layer was washed twice with DCM (2 × 50 mL). The combined organic layers were dried over anhydrous sodium sulfate and concentrated under reduced pressure. The crude product was purified by column chromatography using a methanol/DCM gradient as eluent. The pure final product was obtained with a yield of 61 %.

^{1}H NMR (500 MHz, CD_2Cl_2) δ 4.18 – 4.16 (m, 2H), 3.65 – 3.50 (m, 45H), 3.33 (s, 3H), 2.31 (t, 2H), 1.62-1.56 (m, 2H), 1.29 – 1.25 (m, 18H), 0.87 (t, 3H); **^{13}C NMR (126 MHz, CD_2Cl_2)** δ 173.56, 71.88, 70.53, 70.50, 70.46, 70.37, 69.12, 63.35, 58.63, 53.91, 53.69, 53.48, 34.10, 31.93, 29.67, 29.64, 29.62, 29.50, 29.36, 29.30, 29.12, 24.91, 22.70, 13.91ppm.

Synthesis of C12-triazole-550 (3)

PEG-550-N_3 (1g, 1 eq) and 1- tetradecyne (0.42g, 1,2 eq) was dissolved in 40 mL THF-water mixture (3:1, v/v%) in a 100 mL round bottom flask. After dissolution of the compound, (0.09, 0.2 eq) of copper sulphate were added followed by (0.14g, 0.4eq) of sodium ascorbate. The reaction mixture and was stirred for 24 h at 50 °C The progress of the reaction was monitored by thin layer chromatography (TLC). After completion of the reaction, the THF was removed under reduced pressure and the crude residue was extracted with dichloromethane (DCM) and water. The aqueous layer was washed twice with DCM (2 × 50 mL). The combined organic layers were dried over anhydrous sodium sulfate and concentrated under reduced pressure. The crude product was purified by

column chromatography using a methanol DCM gradient as eluent. The pure final product was obtained with a yield of 92 %

^{1}H NMR (700 MHz, CD_2Cl_2) δ 7.45 (s, 1H), 4.47 (m, 2H), 3.84 (m, 2H), 3.60 – 3.49 (m, 44H), 3.33 (s, 3H), 2.66 (t, 2H), 1.66-1.62 (m, 2H), 1.36 – 1.26 (m, 18H), 0.88 (t, 3H). **^{13}C NMR (176 MHz, CD_2Cl_2)** δ 148.08, 121.60, 71.90, 70.51, 70.38, 69.59, 58.60, 50.02, 31.91, 29.67, 29.61, 29.56, 29.42, 29.34, 29.29, 25.65, 22.68, 13.87ppm.

Synthesis of C12-amide-550 (4)

PEG-550-NH_2 (1.0 g, 1.0 equiv.) was dissolved in 40 mL dry DMF in a 100 mL round bottom flask. 1-dodecanoic acid (0.42 g, 1.2 equiv.) was added, followed by the addition of EDC-HCl (0.42 g, 1.2 equiv.) and HOBt (0.27 g, 1.1 equiv.). The reaction mixture was stirred at 50 °C for 24 hours. The progress of the reaction was monitored by thin layer chromatography (TLC). After completion of the reaction, the DMF was removed under reduced pressure and the crude residue was extracted with dichloromethane (DCM) and water. The aqueous layer was washed twice with DCM (2 × 50 mL). The combined organic layers were dried over anhydrous sodium sulfate and concentrated under reduced pressure. The crude product was purified by column chromatography using a methanol DCM gradient as eluent. The pure final product was obtained with a yield of 65 %.

^{1}H NMR (500 MHz, CD_2Cl_2) δ 3.58 – 3.48 (m, 44H), 3.39 – 3.35 (m, 2H), 3.32 (s, 2H), 2.30-2.10 (m, 2H), 1.61 – 1.55 (m, 2H), 1.28-1.24 (m, 16) 0.86 (s, 3H). **^{13}C NMR (126 MHz, CD_2Cl_2)** δ 173.09, 71.88, 70.49, 70.42, 70.36, 70.22, 69.88, 58.62, 39.14, 36.52, 33.82, 31.93, 29.69, 29.66, 29.64, 29.62, 29.56, 29.50, 29.44, 29.37, 29.32, 29.31, 29.12, 25.76, 24.83, 22.70, 13.90ppm.

NMR data of final detergents

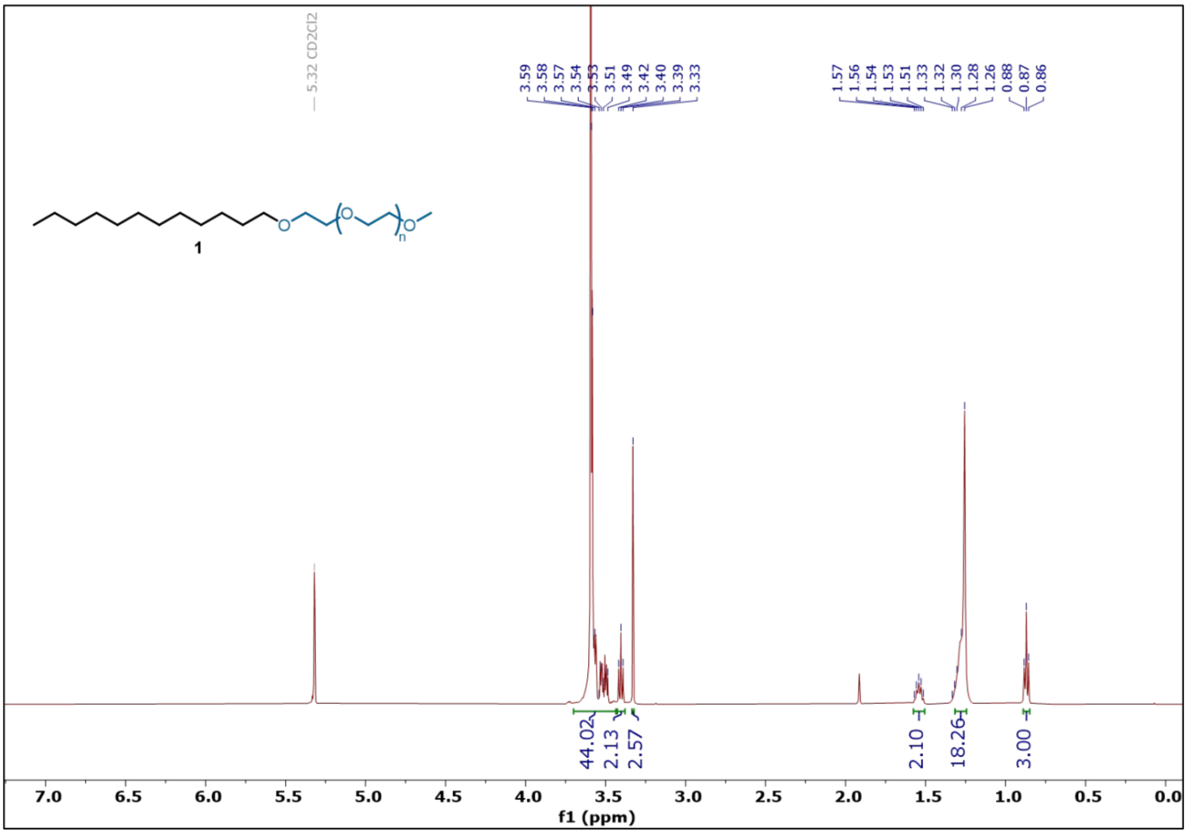

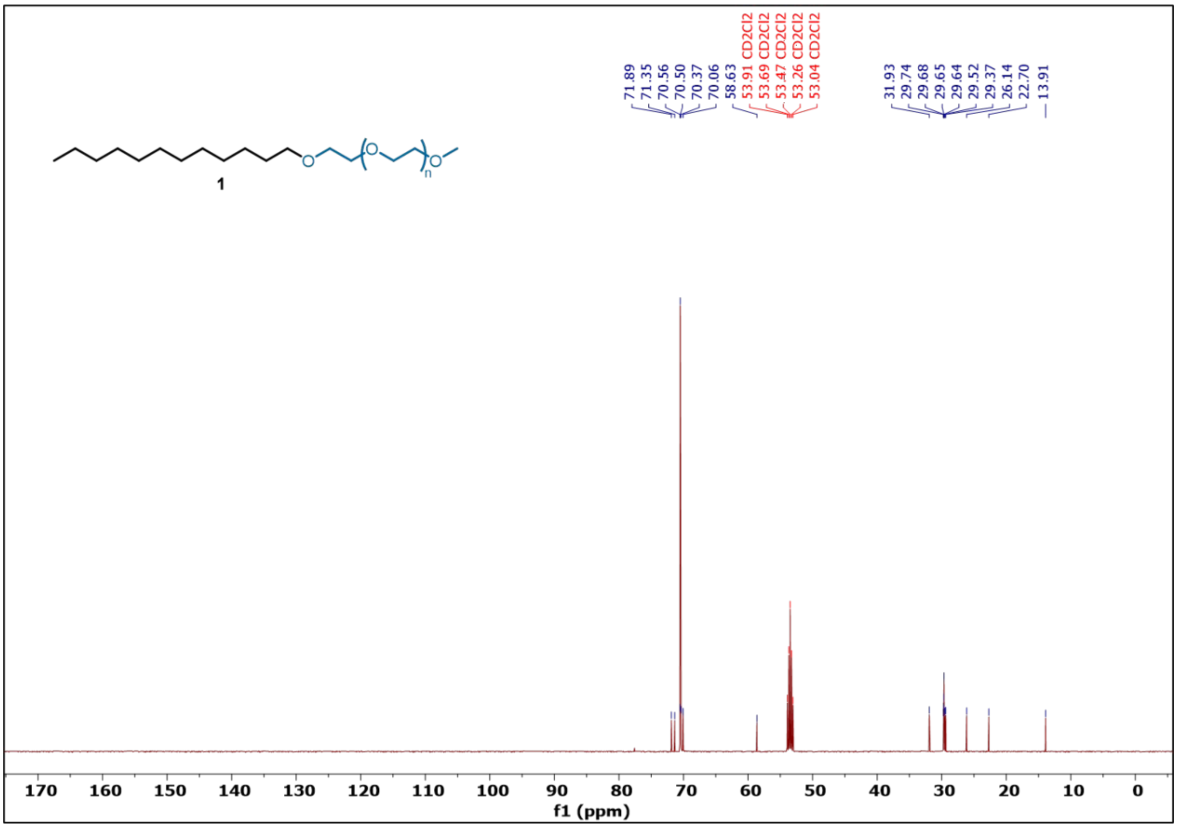

Figure S12: ^{1}H and ^{13}C NMR of detergent **1**.

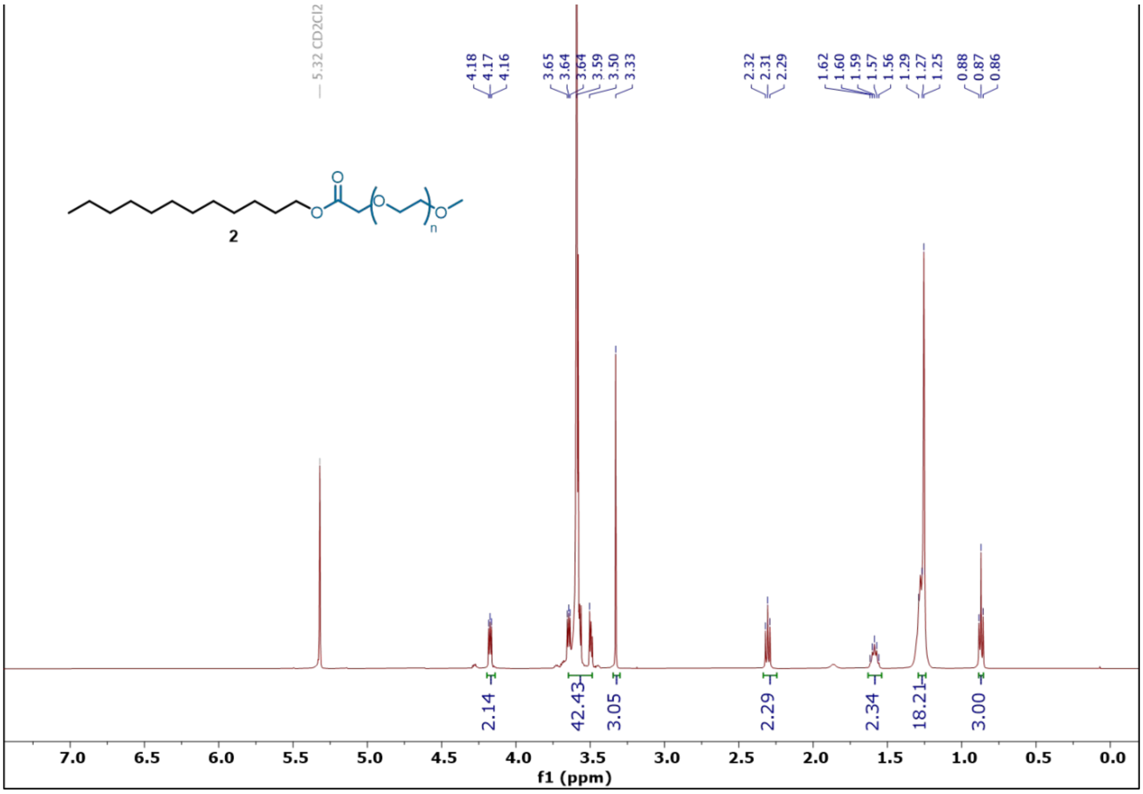

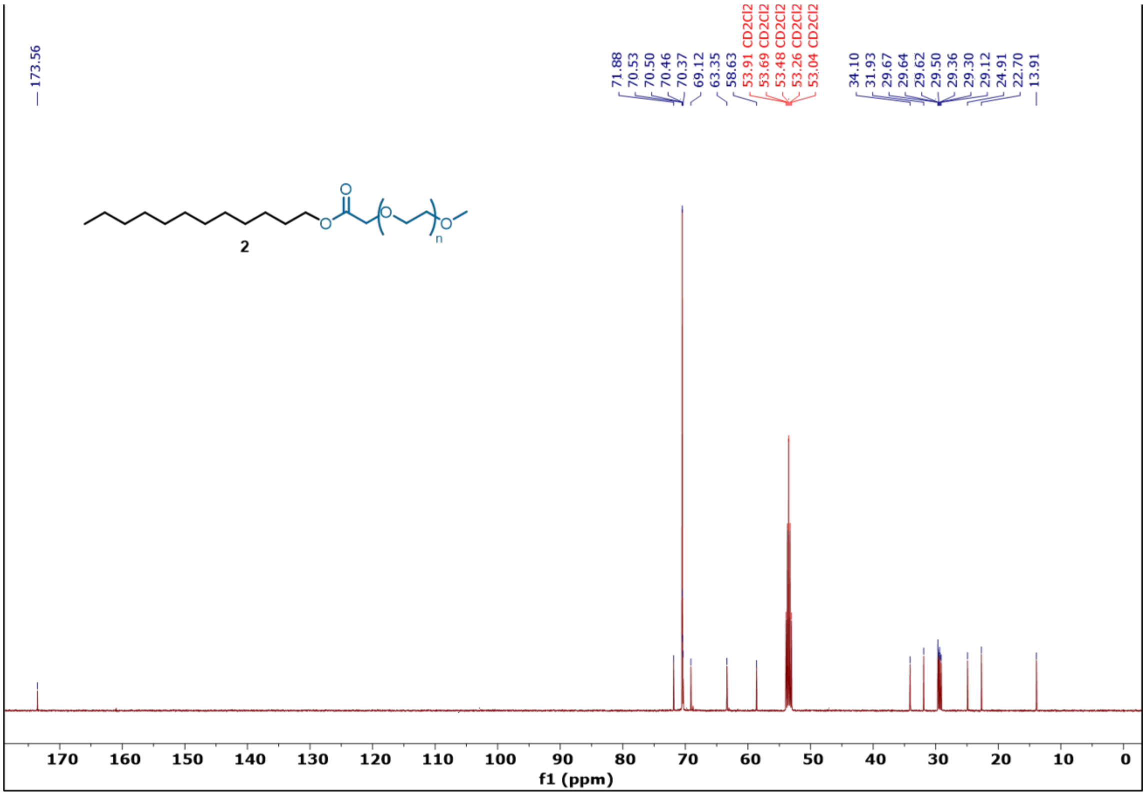

Figure S13: ^{1}H and ^{13}C NMR of detergent **2**.

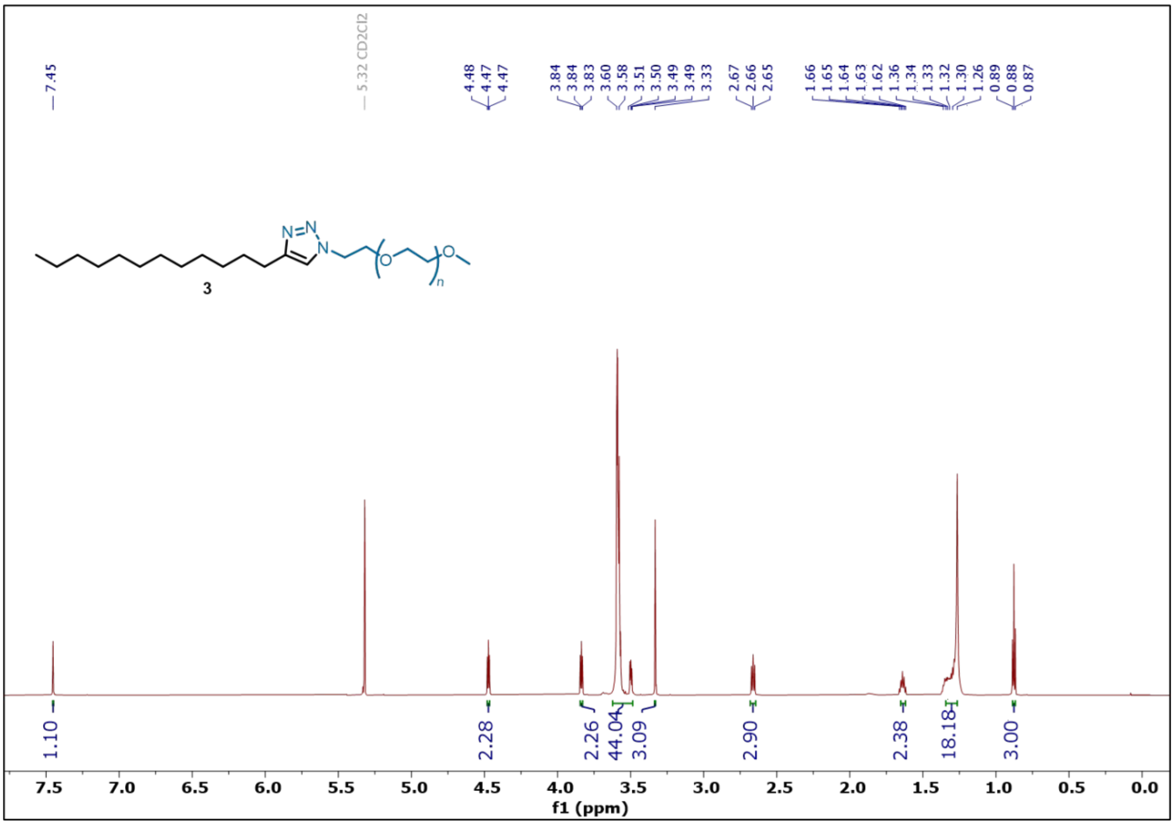

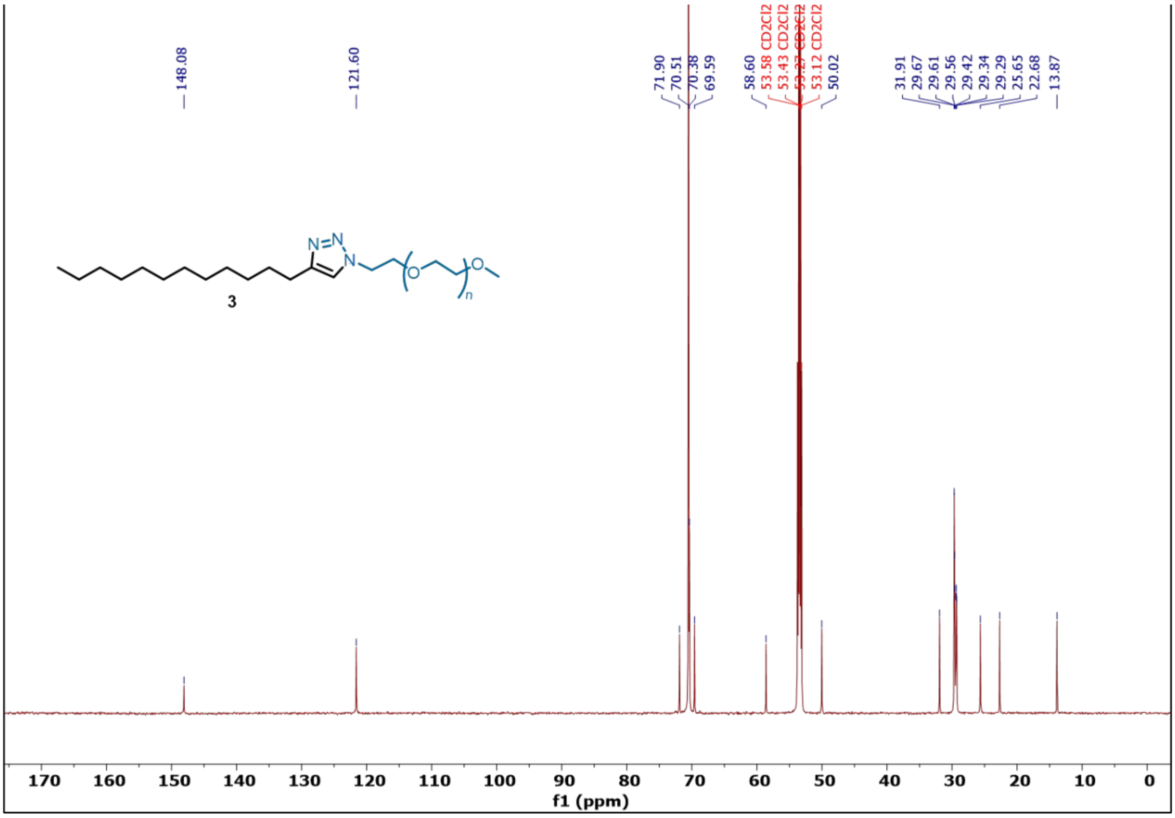

Figure S14: ^{1}H and ^{13}C NMR of detergent **3**.

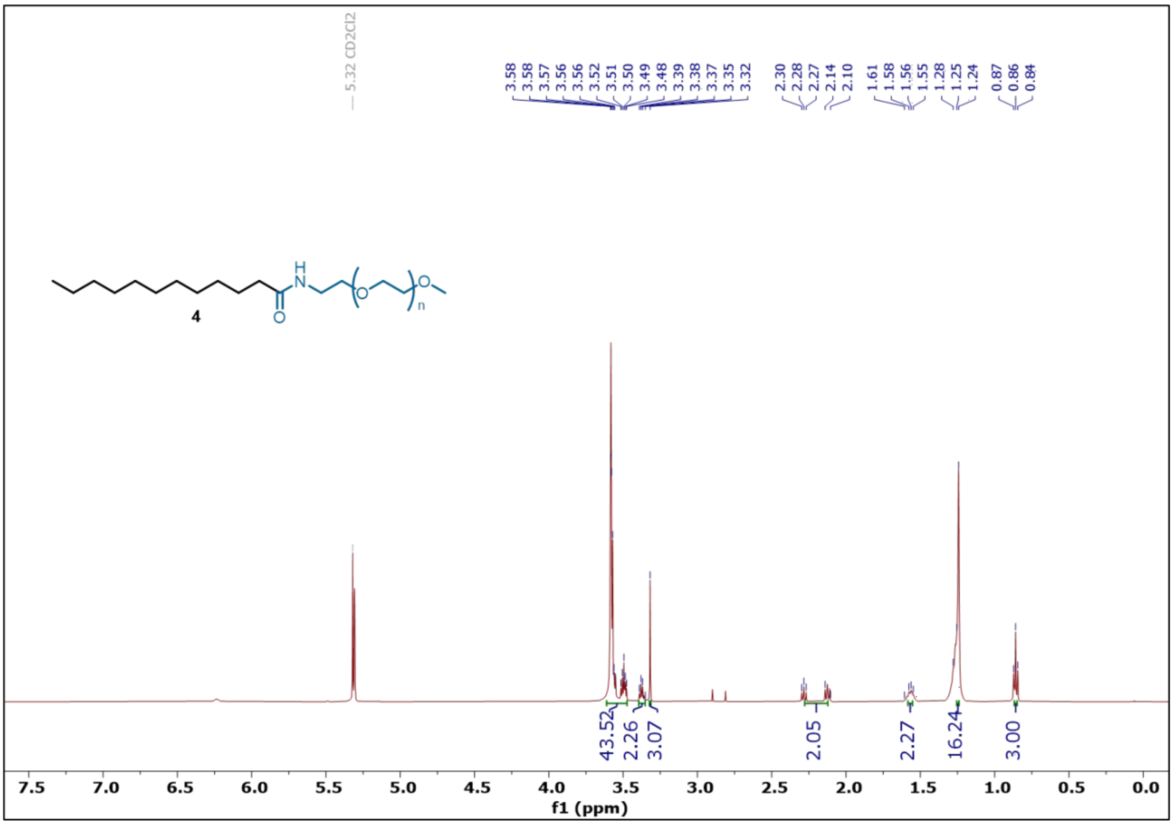

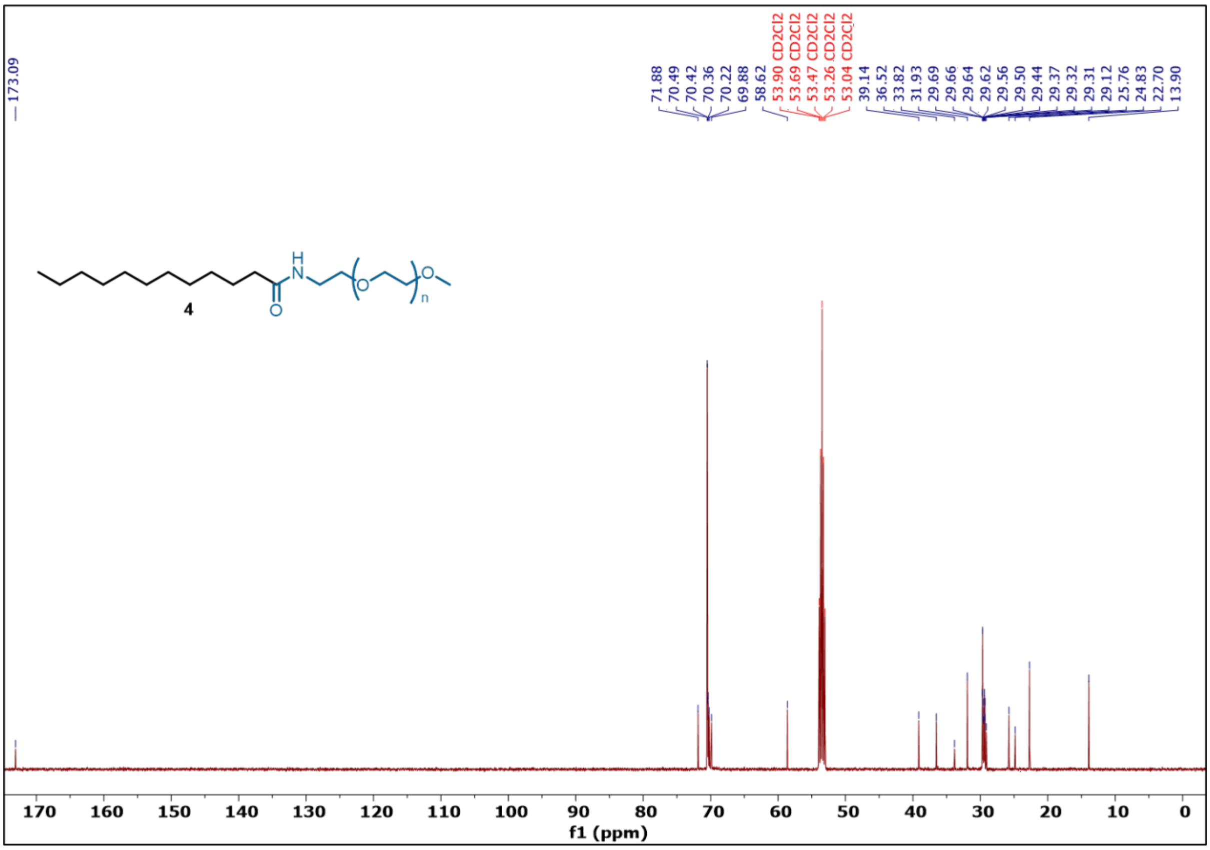

Figure S15: ^{1}H and ^{13}C NMR of detergent **4**.

Experimental procedure Activity Assay

The following protocol of the preparation of BtuCD for the ATPase activity assay, based on the report by Urner *et al.*[1]

All steps of the protein purification procedure were conducted on ice. In a 15 mL falcon tube extraction buffer (7 mL of 200 mM NaCl, 20 mM Tris, pH = 8) was mixed with protein-containing membrane suspension (2 mL) and DDM stock solution (1 mL of 10% w/v aqueous solution). The mixture was inverted and stored on ice for 2 min. The protein-containing supernatant was separated by centrifugation (4000 g, 10 minutes, 4°C). HisTrap HP column (cytiva, column volume 1 mL) was washed with deionized water (2.5 CV) and equilibrated with loading buffer (1 CV of 200 mM NaCl, 20 mM Tris, 20 mM imidazole, DDM 2x cac, pH = 8). The supernatant (7 mL) was applied slowly via syringe onto the column. The column was washed with loading buffer (3 CV) and wash buffer (6 CV of 200 mM NaCl, 20 mM Tris, 40 mM imidazole, DDM 2x cac, pH = 8). A 1.5 mL Eppendorf tube was prepared to collect the purified protein after loading elution buffer (1.5 CV 200 mM NaCl, 20 mM Tris, 200 mM imidazole, DDM 2x cac, pH = 8). The eluate was concentrated to a final volume of 0.5 mL. A desalting spin column (CV = 5 mL, Cytiva HiTrap Desalting, product number: GE17-1408-01) was washed with deionized water (3 CV) and equilibrated with assay buffer (5 CV of 40 mM Tris, 80 mM NaCl, 50 µM EDTA, DDM 2x cac, pH = 8) The concentrated eluate was applied onto the column using a syringe. Assay buffer (1 CV) was used to elute the protein. Fractions were collected (fraction size = 1 mL) and protein-containing fractions were identified by UV spectroscopy, combined, and concentrated to a final protein concentration of 35 µM.

To monitor protein activity, ATPase/GTPase activity assay kit (product number: MAK113 by Sigma-Aldrich) was utilized. Prior to use, provided assay buffer (40 mM tris, 80 mM NaCl, 8 mM $MgAc_2$, 1 mM EDTA, pH = 7) and the protein solution were tested for free phosphate. To this end, 34.3 µL of assay buffer were transferred into a well of a 96-well plate (Greiner, flat bottom transparent polystyrene) and mixed with 5.7 µL of a 35 µM BtuCD solution and 200 µL of provided colorimetric reagent. The mixture was incubated for 30 min and absorbance at 620 nm was measured using a plate reader (TECAN, INFINITE 200 PRO Reader Family). After 30 min, absorbance was lower than 0.3, indicating no free phosphate contamination was present in neither the assay buffer nor the protein solution.

All assay components were allowed to warm up to room temperature. For preparation of a phosphate standard curve, samples containing 0, 15, 30 and 50 µM phosphate were prepared by mixing with the provided phosphate standard according to the manual provided with the kit. A volume of 40 µL of each phosphate concentration was transferred into separate wells of the 96-well plate. For the preparation of the protein samples diluted in PEG550 detergents, 24.3 µL provided assay buffer were submitted and mixed with DDM or PEG550 **1-4** (4x cac) and 5.7 µL of the 35 µM protein stock. For each detergent 40 µL of provided assay buffer (4x cac of DDM or PEG **1-4**) were transferred into separate wells serving as blanks.

After adding 10 µL of a 4 mM aqueous solution of ATP (provided with the kit) to the protein-containing wells, samples were incubated for 30 min at room temperature. After incubation, 200 µL of colorimetric reagent and 20 µL of a 10% w/w solution of Triton X-100 were added to each well, including blanks and phosphate standards. All samples were diluted 1:1 by adding 130 µL assay buffer (40 mM tris, 80 mM NaCl, 8 mM $MgAc_2$, 1 mM EDTA, pH = 7). After 30 min, absorbance at 620 nm of each diluted sample was recorded using a plate reader (TECAN, INFINITE 200 PRO Reader Family).

Calculation of BtuCD activity

To calculate the concentration of free phosphate in protein containing samples a standard curve was created. The 0 µM phosphate sample was used as blank and the absorbance at 620 nm was subtracted from the remaining phosphate standard sample. The blank corrected values were plotted against the phosphate concentration, and a linear regression was performed on the data points to calculate the standard curve. With the help of the resulting formula the concentration of phosphate $c(P_i)$ in protein containing samples was calculated according to

$$c(P_i) = \frac{\Delta P_{620\,\mathrm{nm}} \cdot 2 - b}{m} \tag{1}$$

with $\Delta P_{620\,\mathrm{nm}}$ being the blank corrected absorbance of protein containing samples, m being the slope of the linear fit of the standard curve and b being the y-axis intercept of the linear fit of the standard curve. The activity of BtuCD was then calculated according to

$$activity\ [\mathrm{\mu mol_{P_I}/min/\mu mol_{BtuCD}}] = \frac{n(P_i)}{t \cdot n(P)} \tag{2}$$

With $n(P_i)$ being the molar amount of phosphate, t being the incubation time for the enzymatic reaction and $n(P)$ being the molar amount of BtuCD added to the samples.

SEC elution profile

SEC elution profile was recorded using on an ÄKTA pure chromatography system by Cytiva. BtuCD was transferred from elution buffer to ammonium bicarbonate buffer over a 5 mL SEC column (Cytiva HiTrap Desalting columns, CV = 5 mL, product number: GE17-1408-01). The column was equilibrated with an Äkta setup that was operated at 4 °C. The Äkta was equipped with a sample fractionator and the chromatogram was monitored with a UV/VIS detector at 280 nm. The column was washed with 3 CVs of deionized water and equilibrated with 5 CVs ammonium bicarbonate (100 mM, 2x cac of DDM or detergent **1,** pH = 8). The previously with DDM purified BtuCD was injected into the SEC column. The protein was eluted over 1 CV of detergent-containing ammonium bicarbonate at a flow rate of 5 mL/min.

References

(1) Urner, L. H.; Mohammadifar, E.; Ludwig, K.; Shutin, D.; Fiorentino, F.; Liko, I.; Almeida, F. G.; Kutifa, D.; Haag, R.; Robinson, C. V. Anionic Dendritic Polyglycerol for Protein Purification and Delipidation. *ACS Appl. Polym. Mater.* **2021**, 3, 5903–5911.
